# Evaluating Community-Level Effects of 6PPD-Quinone in a Natural Boreal Lake Ecosystem

**DOI:** 10.1021/acs.est.6c08085

**Published:** 2026-10-16

**Authors:** Erin J. Ussery, Kerri Lynn Ackerly, Kathleen J. Roark, Jose Luis Rodriguez-Gil, Lauren Timlick, Vince Palace, Alon R. Agua, Michael Pirrung, Kristin Nielsen

**Affiliations:** † 514845 Environment and Climate Change Canada, Burlington, Ontario L7S 1A1, Canada; ‡ Department of Marine Science, 12330 The University of Texas at Austin, Port Aransas, Texas 78373, United States; § International Institute for Sustainable Development—Experimental Lakes Area, Winnipeg, Manitoba R3B 0X7, Canada; ∥ Department of Chemistry, 8790 University of California Riverside, Riverside, California 92521, United States

**Keywords:** 6PPD-quinone, mesocosm, limnocorral, developmental toxicity, community effects

## Abstract

6PPD-quinone (6PPD-Q), a transformation product of the tire rubber antioxidant 6PPD, is an emerging contaminant known for its acute toxicity to certain salmonids. However, its potential impacts on aquatic communities remain poorly understood. To address this knowledge gap, 6PPD-Q-spiked limnocorrals were deployed in a natural boreal lake, and water chemistry, phytoplankton, zooplankton, and benthic invertebrate communities were assessed over 28 days following a single pulse exposure (0–100 μg/L) simulating stormwater runoff. Waterborne 6PPD-Q concentrations declined by 80–99% by the end of the study, with no detectable effects on water chemistry or aquatic communities. Temporal changes in chlorophyll-a concentrations and zooplankton abundance were attributed to seasonal dynamics rather than 6PPD-Q exposure, and benthic invertebrate community composition remained unchanged. Concurrently, wild-spawned fathead minnow (*Pimephales promelas*) embryos were exposed to 6PPD-Q-spiked water collected from the limnocorrals. Larval survival declined significantly at 100 μg/L, with altered hatch timing and sublethal effects including reduced growth, changes in eye, yolk sac, and pericardial areas, and altered energy allocation. These effects occurred at 10.6–22.4 μg/L and above, concentrations that exceed those so far reported in surface waters and stormwater by approximately an order of magnitude. Collectively, this study provides the first field-scale assessment of 6PPD-Q effects on aquatic communities and early life-stage fish. Environmental Significance. This study provides the first field-scale assessment of 6PPD-Q impacts on freshwater communities. While broad aquatic communities appeared resilient to short-term exposure scenarios, early life stages of fish responded at concentrations above those currently reported in the environment, identifying developmental end points that warrant attention when assessing the risks of tire-derived contaminants in freshwater ecosystems.

## Graphical abstract

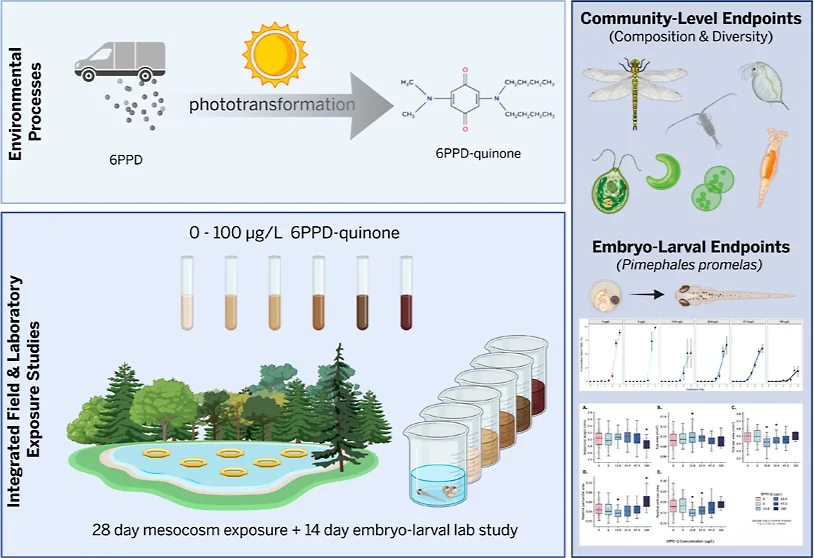

## 1 Introduction

Urban stormwater acts as a major conduit for pollutants, including tire-wear-derived compounds, transporting them through freshwater systems and into receiving waters, including the Great Lakes and many other aquatic ecosystems across North America. Among these contaminants, 2-((4-methylpentan-2-yl)­amino)-5-(phenylamino)­cyclohexa-2,5-diene-1,4-dione (6PPD-Q) has recently emerged as a contaminant of significant concern. This compound forms through the environmental oxidation of 6PPD, an antiozonant and antioxidant widely used in vehicle tires. The identification of 6PPD-Q as the chemical agent responsible for “urban runoff mortality syndrome” in coho salmon (*Oncorhynchus kisutch*) has catalyzed a growing body of research investigating its occurrence, environmental fate, and toxicological effects across a range of aquatic organisms.^1–3^

Reported concentrations of 6PPD-Q during wet-weather flows typically range from 5.6 to 82.3 ng/L (0.0056–0.0823 μg/L),^4^ consistent with levels observed in urban streams in Seattle (<7–210 ng/L; <0.007–0.2 μg/L) and San Francisco (65–230 ng/L; 0.065–0.23 μg/L).^2^ However, substantially higher concentrations (<3.0 μg/L) have been documented in roadway runoff and stormwater-impacted systems along the U.S. West Coast and Ontario, Canada.^2,4,5^ Together these observations highlight the potential for episodic high-exposure events in receiving waters well beyond the West Coast, including systems hydrologically comparable to those of the Boreal Shield region of Canada.

Importantly, sensitivity to 6PPD-Q varies substantially among fishes, and even among closely related salmonids. Coho salmon remain among the most sensitive species tested to date (LC50 of 0.095 μg/L),^2^ and coastal cutthroat trout (*Oncorhynchus clarkii clarkii*) exhibit comparable sensitivity,^6^ indicating that coho salmon are not uniquely at risk at concentrations measured in North American surface waters. Sensitivity is not uniform across cutthroat subspecies, however, and this finding should not be generalized beyond the coastal form. Other salmonids are sensitive at concentrations that nonetheless exceed those typically measured in the environment, including white-spotted char (*Salvelinus leucomaenis pluvius*; 24-h LC_50_ of 0.51 μg/L),^7^ brook trout (*Salvelinus fontinalis*; 24-h LC_50_ of 0.59 μg/L),^8^ early life-stage lake trout (*Salvelinus namaycush*),^9^ and rainbow trout (*Oncorhynchus mykiss*; 24-h LC_50_ of 1.96 μg/L, declining to 1.00 μg/L after 72–96 h).^8^ The sensitivity of lake trout is of particular relevance to boreal and Laurentian Great Lakes systems such as the one examined here. By contrast, several species are considerably less sensitive. Hiki and Yamamoto reported no lethality in southern Asian Dolly Varden (*Salvelinus curilus*) or masu salmon (*Oncorhynchus masou masou*) at any concentration tested,^7^ and Brinkmann et al. observed no mortality in juvenile Arctic char (*Salvelinus alpinus*) after 96 h at measured concentrations as high as 14.2 μg/L.^8^ Similarly, neither brown trout (*Salmo trutta*) nor Atlantic salmon (*Salmo salar*) showed acute lethality at concentrations up to 12.6 μg/L.^10^

Among fishes, nonsalmonid species generally exhibit substantially lower sensitivity. Concentrations as high as 34 μg/L and 54 μg/L did not cause acute lethality in zebrafish (*Danio rerio*) and Japanese medaka (*Oryzias latipes*), respectively.^11^ Similarly, no mortality occurred in juvenile white sturgeon (*Acipenser transmontanus*) exposed to concentrations as high as 12.7 μg/L for 96 h,^8^ and red drum (*Sciaenops ocellatus*) exhibited an LC_50_ of >500 μg/L.^1^ Most literature to date has primarily focused on the acute lethality of 6PPD-Q, with few studies focusing on sublethal effects and mechanistic drivers of toxicity. However, work by Anderson-Bain et al., 2023 observed that 6PPD-Q may disrupt one-carbon metabolism and cause oxidative stress in both the gills and livers of lab-reared adult fathead minnows (*Pimephales promelas*), while 6PPD-Q did not cause malformations or hatching delays of lab-reared embryonic fathead minnows at concentrations from 0.11 to 39.27 μg/L.^12^ Among the limited data from invertebrates, the lowest observed effect concentration (LOEC) of *Hexagenia* spp., *Daphnia magna, Planorbella pilsbyri*, and *Megalonaias nervosa* was found to be 11.4 μg/L while no acute toxicity in *D. magna* or the amphipod *Hyalella azteca* was observed in another study.^11,13^

Although the body of literature on the effects of 6PPD-Q in single-species assays has grown substantially, studies evaluating its impacts at the whole-ecosystem level remain limited. Therefore, the present study aimed to investigate the fate and ecological effects of 6PPD-Q using a whole-ecosystem approach under semicontrolled field conditions. To capture relevant environmental processes while minimizing the uncertainty commonly associated with conventional field studies, we employed an in-lake mesocosm design, providing a balance between ecological realism and experimental control to investigate the toxicological effects of 6PPD-Q on early life stage (ELS) wild fathead minnow, periphyton abundance, zooplankton, and benthic invertebrate communities.

## 2 Materials and Methods

All experimental protocols were approved by The University of Texas at Austin Institutional Animal Care and Use Committee (IACUC; AUP-2021-00078) and the University of Manitoba Fort Garry Campus Animal Care Committee (F21-005).

### 2.1 Study Area

Mesocosms were deployed at the International Institute for Sustainable Development—Experimental Lakes Area (IISD-ELA), southeast of Kenora in the Boreal Shield ecoregion (Ontario, Canada). Mesocosms were deployed on the southern edge of Lake 302, a boreal lake with a surface area of 233,161 m^2^, a total volume of 1,277,471.63 m^3^ and a maximum depth of 14.03 m.^14^ Lake 302 was chosen for this study because of the abundance of historical data archived for this lake as well as numerous published limnocorral data and the availability of large areas with sediment at the 1.5–2 m depth.^15–19^

### 2.2 Limnocorral Study Design

Mesocosms (*n* = 8) consisting of an impermeable polypropylene curtain (Curry Industries, Winnipeg, Canada) suspended from a floating ring (2-m diameter; 1.5–2-m depth), were deployed in Lake 302 at the IISD-ELA in June of 2023. Mesocosms were randomly assigned treatments of 0, 5, 10.6, 22.4, 47.3, or 100-μg/L 6PPD-Q (Figure 1). Polypropylene curtains were sealed to the floor of the lake by placing sandbags around the exterior wall, creating a representative sample of the natural boreal lake system that included the sediment, water column (approximately 6000-L per mesocosm), and associated invertebrate biota. Any incidentally entrained fish were removed from limnocorrals with minnow traps prior to study commencement and each structure was covered with predator mesh (1 × 1 in.) to prevent treatment-independent impacts to mesocosm food webs.

**Figure 1:**
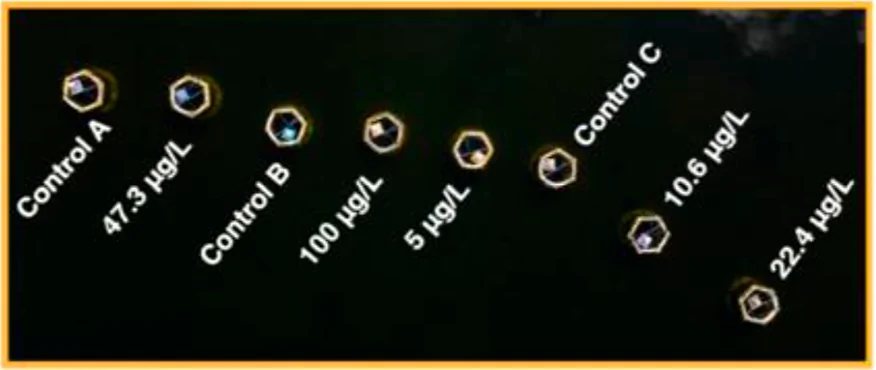
Experimental mesocosm and field deployment. Aerial photograph of the mesocosm array deployed in Lake 302, showing the eight mesocosms used in this study. The mesocosms were randomly assigned to three controls and five 6PPD-Q concentration treatments (5, 10.6, 22.4, 47.3, and 100 μg/L).

Temperature (°C), dissolved oxygen (DO; mg/L), and light intensity (lux) were continuously monitored in all limnocorrals using HOBO data loggers (Onset Computer Corporation, Bourne, MA, USA) for 2 weeks prior to study commencement, and for the duration of the study itself (daily averages are presented in Table S1). Logger data was manually corroborated once daily with a hand-held YSI ProQuattro probe (YSI Inc., Yellow Spring, OH, USA), along with collection of additional parameters including pressure (kPa), specific conductivity (μS/cm), and pH.

At the conclusion of the 2-week baseline monitoring period, a commercially produced 6PPD-Q standard (97.26% purity; Item #: 687855; HPC Standards, Atlanta, GA, USA) was added to limnocorrals to achieve one of the following target concentrations: 0, 5, 10.6, 22.4, 47.3, or 100-μg/L (Table 1). A 3.33 mg/mL stock solution was made on 26 June 2023 by dissolving 6PPD-Q in a combination of 4.75 mL dimethyl sulfoxide (DMSO), 4.75 mL HPLC-grade methanol, and 1.40 mL acetone. The stock solution was covered in aluminum foil and mixed overnight before being used to prepare spike solutions on 27 June 2023. Mesocosms were spiked with 6PPD-Q on 27 June 2023. Following addition of 6PPD-Q, no active mixing was applied; the compound was allowed to disperse naturally within the water column of the limnocorrals. As water samples were collected using an integrated sampler spanning the entire water column, reported concentrations represent a depth-integrated average rather than a discrete-depth measurement. Treatments were randomly assigned to limnocorrals in the chosen array within Lake 302 (Figure 1) and 6PPD-Q was not renewed further. This approach was used to simulate contamination via stormwater runoff and to allow for an evaluation of the environmental persistence of the compound in a natural system.

**Table 1:** Average (±Standard Error of the Mean) of Nominal Versus Measured Concentrations of 6PPD-Q in In-Lake Mesocosms and Embryo-Larval Laboratory Experiments^a^

initial spike (12 h postspike)	
nominal concentration (μg/L)	measured concentration (μg/L)
5	3.3
10.6	8.2
22.4	18.2
47.3	40.1
100	78.4

final concentration (4-week postspike)	
nominal concentration (μg/L)	measured concentration (μg/L)
0	0.0
5	0.2
10.6	0.2
22.4	1.1
47.3	16.4
100	8.6

final concentrations (embryolarval)	
nominal concentration (μg/L)	measured concentration (μg/L)
0	0.0
5	4.5
10.6	13.8
22.4	31.2
47.3	44.2
100	105.1

a Methods for collection, extraction, and analyses are detailed in the text.

### 2.3 Sample Collection

Water samples were collected during the baseline monitoring period and on study days 1, 14, and 28. Samples were collected using a depth integrated sampler and stored in 200 mL glass amber volumes for analysis of 6PPD-quinone, and to collect data on water chemistry parameters including dissolved inorganic carbon (DIC), dissolved organic carbon (DOC), total dissolved nitrogen (TDN), suspended phosphorus (P), reactive silica (Si), suspended iron (Fe), and sulfate (SO_4_).^50^ Biological end points evaluated in water samples included chlorophyll-*a* (as an indicator of phytoplankton abundance and biomass/photosynthetic potential), ash free dry mass (AFDM), phytoplankton community composition, and zooplankton abundance and community composition. Chlorophyll-*a* was measured once prior to spiking, and once after. AFDM was only measured once post spike. Zooplankton samples were collected using a Wisconsin-type zooplankton net with a 0.25 m diameter opening and a 53 μm mesh, towed vertically through the water column (*n* = 1 per limnocorral prespike, and *n* = 3 per limnocorral postspike) to capture a representative sample across multiple depths. Collection of benthic macroinvertebrates was conducted using modified Hester-Dendy benthic samplers (*n* = 1 per limnocorral prespike, and *n* = 5 per limnocorral postspike), which were deployed at the water–sediment interface.^20^

### 2.4 Embryo-Larval Fish Exposures

Because fathead minnows did not reproduce within the experimental mesocosms, embryos could not be collected directly from the study populations. To evaluate whether mesocosm water retained developmental toxicity after undergoing fate and transformation processes within the environment, wild-collected fathead minnow embryos were exposed to water collected from each mesocosm treatment. On study day 14, an ELS exposure study was initiated following the general methods described in ref 21. Just after sunrise, wild fathead minnow embryos were collected from breeding tiles deployed in Lake 114. Lake 114 was selected to collect fathead minnow embryos due to the availability of extensive historical data on population health and because of its healthy, spawning resident fathead minnow population which have been used in numerous other embryonic exposure studies (e.g., Nielsen et al., 2022), as well as the small population of resident fathead minnows in Lake 302.^21^ Tiles were immersed in a 1% sodium sulfite solution for less than 2 min; detached eggs were then rinsed into a collection vessel prefilled with aerated lake water. Eggs were then immediately transported to an IISD-ELA laboratory, where viability was assessed using a ZEISS Stemi 2000-C stereoscope.

Viable embryos (*n* = 20 per dish) were exposed to 6PPD-Q for 7 days under controlled laboratory conditions (16 h light: 8 h dark), using 250 mL glass jars as exposure chambers. Chambers were prefilled with 200 mL of treatment water (*n* = 3 replicates per treatment) collected from in-lake limnocorrals 12 h following 6PPD-Q spike. Water samples were taken from each mesocosm using an integrated sampler to facilitate sampling the entire water column and held in clean 4-L glass bottles. This single 12-h collection served as the water source for the 7-day exposure period, including the daily 80% water renewals; no additional field water was collected after the initial 12-h postspike time point. Water was brought back to the lab and stored in the dark at 4 °C until the beginning of the embryo-larval laboratory experiment, and for the duration of the exposure period for renewal water. Water was warmed to room temperature (22 °C) and bubbled with air for 30 min prior to the start of the in-lab experiment and prior to each daily renewal. Mortality and hatch rate were assessed daily, just prior to water changes (80%), which were conducted using the same previously collected mesocosm water. Water quality was measured each day immediately prior to water changes (Table 2). Between water changes, mesocosm water was maintained at 4 °C in the dark to prevent potential 6PPD-Q degradation.

**Table 2:** Mean Daily Temperature (°C) and Light Intensity (Lux) Recorded during the 8-Day Embryo-Larval Exposure Period^a^

exposure day	temperature (°C)	light (Lux)
1	21.97 ± 0.04	208.99 ± 14.65
2	19.97 ± 0.02	201.61 ± 14.47
3	19.6 ± 0.07	210.23 ± 12.48
4	20.54 ± 0.02	185.47 ± 11.4
5	20.16 ± 0.04	191.83 ± 12.22
6	20.82 ± 0.04	173.57 ± 11.11
7	19.71 ± 0.03	317.49 ± 19.85
8	18.53 ± 0.03	268.26 ± 41.09

a Values are expressed as mean ± standard error.

On study day 21 (7 DPF), larvae were humanely sacrificed in buffered MS-222 in accordance with the approved methods described in AUP-2021-0225 and F21-005. Immediately thereafter, larvae were placed in 3% isotonic methylcellulose and imaged using a ZEISS Stemi 2000-C stereoscope fitted with a camera. Recorded images were blinded prior to analysis of morphological end points (Figure S1), including the total length, yolk sac area, pericardial area, eye area, swim bladder area, and lateral body surface area (excluding fin surface area). All morphology measurements were conducted with ImageJ (NIH, version 1.51). Relative areas were calculated by dividing the measured tissue area (e.g., brain area) by the whole-body area to get a relative area of that tissue. Specific equations are relative body area = body area/total length; relative brain area = brain area/whole body area; relative pericardium = pericardium area/body area; relative eye area = eye area/body area; relative yolk sac area = yolk sac area/body area; relative swim bladder area = swim bladder area/body area.

### 2.5 Statistical Analysis

Biofilm responses to 6PPD-Q were evaluated using chlorophyll-a concentration and AFDM as complementary indicators of biomass and function. Chlorophyll-*a* was used as a proxy for photosynthetic biofilm biomass, whereas AFDM was used as a measure of total organic biofilm mass, including autotrophic and heterotrophic components.^22,23^ Chlorophyll-*a* samples were measured at two time points representing pre- and postspike conditions. Concentrations were analyzed with a linear model with treatment concentration, spike stage, and their interaction as fixed effects.

AFDM was calculated as the difference between dry mass and ash mass following combustion. Small negative values attributable to analytical precision were set to zero prior to analysis. Because AFDM samples were collected only during the postspike period, AFDM was analyzed as a function of treatment concentration only using a linear model. In addition, Spearman rank correlation was used to test monotonic associations between treatment concentration and AFDM. All analyses were conducted in R,^51^ with significance assessed at α = 0.05.

Phytoplankton were identified down to the lowest practical taxonomic level. Three complementary data sets were analyzed: total biomass, total cell density, and species-level community composition.^24,25^ Total phytoplankton biomass and density were analyzed using linear models with treatment concentration, spike stage, and their interaction as fixed effects. Species-level abundance data were aggregated by sample to construct a sample × species community matrix using biomass estimates. From this matrix, alpha diversity metrics were calculated using the vegan package in R, including taxa richness, Shannon diversity, and Simpson diversity.^26^ These metrics were analyzed using the same linear model structure described above.

Multivariate community composition was evaluated using Bray–Curtis dissimilarities.^27^ Nonmetric multidimensional scaling (NMDS) was used to visualize similarities among samples in reduced ordination space.^28^ Permutational multivariate analysis of variance (PERMANOVA; adonis2) was used to test for effects of treatment concentration, spike stage, and their interaction on phytoplankton community composition using 999 permutations.^29^ Similarity Percentages analysis (SIMPER) was conducted to identify taxa contributing most strongly to differences between pre- and postspike communities.^30,31^

Zooplankton were identified to the lowest practical taxonomic level by Biologica Environmental Services (Victoria, British Columbia). Sampling dates were classified relative to the contaminant addition event (June 27, 2023) as prespike (June 24, 2023) or postspike (all subsequent sampling dates). Life stages and sexes of adults were also identified. Community responses were statistically evaluated using a combination of univariate and multivariate community ecology approaches in RStudio. Raw count data were aggregated by sample (mesocosm × sampling date). Total abundance (raw count multiplied by split multiplier, where applicable), sex ratio, and life-stage proportions were calculated for each sample. Linear models were used to examine relationships between treatment concentration and each response metric while accounting for sampling date as a categorical predictor. Where appropriate, Spearman rank correlations were also used to test for monotonic dose–response relationships independent of distributional assumptions.^32^

For zooplankton, community diversity was quantified using standard alpha-diversity metrics calculated from the sample-by-taxon abundance matrix using the vegan package.^33^ Taxonomic richness was defined as the number of taxa present per sample. Shannon diversity incorporated both richness and relative evenness, and Simpson diversity emphasized dominance structure within samples. Diversity metrics were analyzed using linear models with treatment concentration and sampling date as predictors. Community composition was further evaluated using Bray–Curtis dissimilarities derived from taxon abundance data. NMDS was used to visualize compositional differences among samples, and PERMANOVA (999 permutations) was used to test for associations between community structure, treatment concentration, and sampling date. SIMPER was used descriptively to identify taxa contributing most strongly to observed compositional differences among groups.

Benthic insect communities were identified to the lowest practical taxonomic resolution using standard regional keys,^34^ and counts were recorded for each taxon within each sample. Raw count data were used to evaluate abundance, diversity, and community composition responses to treatment concentration and sampling period. Total aquatic insect abundance per sample was calculated as the sum of all observed individuals across taxa. Taxonomic count data were aggregated by sample to generate a sample × taxon abundance matrix, with absent taxa assigned zero values. From this matrix, alpha diversity metrics were calculated using the vegan package in R, including taxa richness, Shannon diversity, and Simpson diversity. Total abundance and diversity metrics were analyzed using linear models with treatment concentration, spike stage, and their interaction as fixed effects.

Multivariate community composition for benthic insects was assessed using Bray–Curtis dissimilarities derived from the abundance matrix. NMDS was used to visualize similarities among samples in reduced ordination space. PERMANOVA was used to test for effects of treatment concentration, spike stage, and their interaction on aquatic insect assemblage structure using 999 permutations. SIMPER was conducted to identify taxa contributing most strongly to significant differences between pre- and postspike communities. All the above metrics (biofilm, phytoplankton, zooplankton, and benthic insects) were visualized using the *ggplot2*, *scales*, and *patchwork* packages in R Studio.

Statistical analyses for the embryo–larval fish morphological components of the study were performed in R (version 4.4.2; R Core Team) using the RStudio interface. Treatment effects were assessed using linear mixed-effects models, with treatment included as a fixed effect and jar included as a random effect. The contribution of the random effect was evaluated, and where jar did not significantly explain variability, it was removed and models were refit without the random term. Prior to analysis, outliers were identified and removed using Grubbs’ outlier test (*Z*-test). Where significant treatment effects were detected, pairwise comparisons between treatments and the control were conducted using Dunnett’s *post hoc* test. Swim bladder data were log-transformed to meet model assumptions. Figures were generated using the *ggplot2* package.

Final survival and hatch rate data were analyzed using JMP Pro (Version 19). A one-way ANOVA was used to assess differences among doses. Survival data were arcsine transformed prior to analyses. Cumulative hatch rate (% hatched per day) was analyzed using mixed models, with dose and exposure day as fixed effects and jar number nested within dose as random effect. As the random effect did not significantly affect the model, it was removed and only fixed effects were included in the analysis. When significant differences occurred, a Dunnett’s *post hoc* test was used to determine significant differences among doses compared to the control. To note, three control jars were excluded from analyses due to documented fungal contamination, likely originating from the original mesocosm.

### 2.6 Analytical Methods for 6PPD-Q Quantification

Each sample bottle was thoroughly rinsed with 10 mL LCMS grade H_2_O and then dried for 10 min. Water samples collected from each mesocosm and final water samples collected from embryo-larval exposure vessels/chambers were spiked with deuterated 6PPD-Q (D5-6PPD-quinone) to a final concentration of 25 μg/L. Solid phase extraction was subsequently performed using Oasis PRiME HLB extraction cartridges (200 mg, 6 mL).^1^ Briefly, each cartridge was conditioned with 10 mL LCMS grade methanol followed by 10 mL of LCMS grade H_2_O. Unfiltered water samples were then loaded into each cartridge at a rate of 5–10 mL per minute. Cartridges were then stored in aluminum foil at −20 °C until elution and final analyses.

Samples were eluted and analyzed for 6PPD-Q at The University of Texas at Austin Marine Science Institute as described in Ackerly et al., 2024 using high performance liquid chromatograph coupled to a triple-quadruple mass spectrometer (HPLC/MS, Shimadzu LCMS-8045). Each sample was eluted with LCMS grade methanol and concentrated under nitrogen flow before being reconstituted with methanol.^1^ Chromatographic separation was achieved on a Phenomenex Lune 3 μm C18 100 reverse phase column (150 × 3 mm) coupled with a C18 guard column, maintained at 45 °C. Two mobile phases were prepared using LCMS grade solvents: mobile phase A consisted of H_2_O containing 0.1% formic acid (v/v), and mobile phase B was a 1:1 (v/v) blend of methanol and acetonitrile, acidified with 0.1% formic acid (v/v). Samples were introduced at an injection volume of 10 μL with a pump flow rate of 0.2 mL/min the following binary gradient elution program was applied: isocratic at 50% B from 0 to 0.5 min, a linear ramp from 50% to 100% B over 0.5 to 10.5 min, held at 100% B from 10.5 to 12 min, returned to 50% B from 12 to 15 min, and re-equilibrated at 50% B from 15 to 20 min.

Mass spectrometric detection was conducted in positive electrospray ionization (ESI+) mode using multireaction monitoring (MRM). Nitrogen gas served as the nebulizing gas at a flow rate of 3 L/min, while both the heating gas (nitrogen) and drying gas (zero grade air) were set to 10 L/min. The interface and desolvation line (DL) temperatures were each maintained at 300 °C. A dwell time of 400 ms was applied. For 6PPD-Q, MRM transitions of *m*/*z* 299.2 à 215.1 (14 eV) and 299.2 à 187.1 (26 eV) were monitored as the qualitative and quantitative ion transitions, respectively. The D5-6PPD-Q internal standard was tracked using transitions of *m*/*z* 304.2 à 220.1 (14 eV) and 304.2 à 192.1 (26 eV) for quantitative and qualitative analyses. Analyte concentrations were determined by reference to an established calibration curve.

## 3 Results

### 3.1 Analytical Verification of 6PPD-Q in Exposure Solutions

Nominal versus measured 6PPD-Q concentrations for both lake mesocosms and embryo-larval exposures are detailed in Table 1. Table 1 reports measured concentrations of 6PPD-Q spiked mesocosms at two time points: 12 h postspike and 4 weeks postspike. At 12 h postspike, measured concentrations ranged from 3.3 to 78.4 μg/L; percent recoveries ranged from 66.1% to 84.9% (Table 1). No 6PPD-Q was detected in any control mesocosm. By 4 weeks postspike, 6PPD-Q concentrations had substantially declined among all treatment levels. Measured concentrations ranged from 0.2 μg/L to 16.4 μg/L, with an average decline in 6PPD-Q concentrations of 87.01% (Table 1). Again, no 6PPD-Q was detected in any of the control mesocosms. Final 6PPD-Q concentrations are shown for water collected at the conclusion of the embryo-larval laboratory exposure experiments (Table 1). Measured concentrations ranged from 4.5 μg/L to 117.3 μg/L across all nominal doses, with percent recoveries ranging from 89.9% to 139.4% (Table 1). No 6PPD-Q was detected in control replicates (Table 1).

### 3.2 Effects on Photosynthetic Potential and Phytoplankton Communities

Biofilm chlorophyll-*a* concentrations were significantly higher postspike when compared to prespike values (estimate = +134.5 μg/L, *p* < 0.001; Figure S2). Chlorophyll-*a* was not significantly associated with 6PPD-Q concentration (*p* = 0.243), and the interactive effects between concentration and sampling period were not significant (*p* = 0.690). AFDM also did not have any significant correlation with the concentration of 6PPD-Q (*p* = 0.353; Figure S3). Spearman correlation analysis further confirmed no monotonic relationship between concentration and AFDM (ρ = 0.116, *p* = 0.448). Total biomass of phytoplankton was significantly lower following the spike period (estimate = −43.4, *p* = 0.044), but this was not significantly associated with 6PPD-Q concentration (*p* = 0.860). There was no significant interaction between concentration and spike stage (*p* = 0.358). Total phytoplankton density was not significantly associated with 6PPD-Q concentration (*p* = 0.301) and did not differ significantly between pre- and postspike periods (*p* = 0.548). The interactive effects between concentration and spike stage were not significant (*p* = 0.067).

PERMANOVA found that phytoplankton community composition was not significantly associated with 6PPD-Q concentration (*R* ^2^ = 0.031, *p* = 0.339; Figure S4A). There were also no significant interaction effects between concentration and spike stage (*R* ^2^ = 0.021, *p* = 0.730). In contrast, community composition differed significantly between pre- and postspike sampling periods (*R* ^2^ = 0.158, *p* = 0.001). Complementary SIMPER analysis found that the pre- to postspike shift in phytoplankton community composition was driven primarily by decreases in several chrysophyte and chlorophyte taxa, including *Chrysochromulina laurentiana*, *Sphaerocystis schroeteri*, *Uroglena volvox*, and *Dinobryon sertularia*. *Merismopedia tenuissima* increased postspike and was the third-largest contributor to community dissimilarity.

Phytoplankton taxa richness was not significantly associated with 6PPD-Q concentration (*p* = 0.891), spike stage (*p* = 0.496), or their interaction (*p* = 0.709) Figure S4B. Shannon diversity increased significantly following the spike period (estimate = +0.208, *p* = 0.002). However, Shannon diversity was not significantly associated with 6PPD-Q concentration (*p* = 0.732), and no interactive effect between concentration and spike stage was detected (*p* = 0.722) (Figure S4C). Simpson diversity increased significantly following the spike period (estimate = +0.027, *p* = 0.005), but this was also not significantly associated with 6PPD-Q concentration (*p* = 0.429), and there were no interactive effects between concentration and spike stage (*p* = 0.588) (Figure S4D).

### 3.3 Effects on Zooplankton Communities

NMDS (stress = 0.194) indicated temporal shifts in zooplankton community composition across the experiment (Figure S5A). Total zooplankton abundance showed no relationship with treatment concentration (linear regression: β = 0.37, *p* = 0.97, *R* ^2^ < 0.016) and did not differ between pre- and postspike conditions (*p* = 0.55) (Figure S5A,B). Similarly, no monotonic association between dose and abundance was detected (Spearman’s ρ = 0.01, *p* = 0.96). In contrast, abundance varied significantly across sampling dates (*p* < 0.001), indicating temporal rather than treatment-driven patterns (Figure S5B). No relationship between treatment concentration and sex ratio was detected (linear regression: β = −0.00018, *p* = 0.16) and sex ratios remained consistent between pre- and postspike sampling periods (*p* > 0.1), indicating stability in population structure over time (Figure S5D).

No significant relationship between treatment concentration and adult proportion was observed (β = 0.00018, *p* = 0.324) and adult proportions did not differ between pre- and postspike conditions (*p* > 0.18) (Figure S5C). Sampling date also did not significantly influence adult proportion (all *p* > 0.18). Overall model fit was low (adjusted *R* ^2^ < 0), indicating limited explanatory power of dose and time. As nauplii are known to be a sensitive life stage, the relationship between nauplii and treatment was also assessed for further exploration. No significant relationship between treatment concentration and nauplii proportion was detected (β = 0.00073, *p* = 0.255). Sampling date also did not significantly influence nauplii proportion overall, although a marginal increase was observed on 13 July 2023 relative to the initial sampling date (β = 0.099, *p* = 0.096). The model explained limited variation in nauplii proportion (adjusted *R* ^2^ = 0.09), suggesting that neither treatment nor sampling date strongly structured this end point. Similarly, Spearman rank correlation indicated no significant monotonic association between dose and proportion of females (ρ = −0.20, *p* = 0.29). Sex ratios were also consistent across sampling dates (*p* > 0.1), indicating stability in population structure over time (Figure S5D).

PERMANOVA additionally signaled a significant effect of sampling date on community structure (*R* ^2^ = 0.1, *p* = 0.002). Treatment concentration alone was not significant (*R* ^2^ = 0.008, p = 0.978). When both predictors were included, sampling date remained highly significant (*p* = 0.001) and treatment concentration approached significance (*p* = 0.0552). SIMPER analysis indicated that treatment-associated differences in zooplankton community composition were driven primarily by cyclopoid copepods (*Cyclopoida* indet.), *Diaphanosoma birgei*, *Bosmina longirostris*, and *Microcyclops rubellus*. Contrasts involving the 47.29 μg/L treatment exhibited the greatest compositional divergence.

Zooplankton diversity metrics were influenced primarily by sampling date rather than treatment concentration. Taxonomic richness did not vary with dose (*p* = 0.56) and did not differ significantly between pre- and postspike conditions (*p* = 0.23). However, richness increased significantly by the final sampling date (β = 3.79, *p* < 0.001). Shannon diversity was not significantly associated with treatment concentration (*p* = 0.61) or spike stage (*p* = 0.11), although a modest increase was observed following the spike period (Figure S6A). In contrast, Simpson diversity increased significantly postspike (β = 0.067, *p* = 0.018; Figure S6B), while remaining unrelated to treatment concentration (*p* = 0.62), further supporting short-term shifts in taxon dominance followed by recovery to a more even community structure.

### 3.4 Effects on Benthic Invertebrate Communities

Total aquatic insect abundance did not vary significantly because of exposure to 6PPD-Q, the spike stage, or the interaction of the two. While postspike abundance tended to be higher than prespike abundance in the control group, this pattern was not statistically significant, and none of the dose-specific postspike contrasts differed significantly from the control response. Overall model fit was weak (adjusted *R* ^2^ = 0.073), indicating that the dose × spike stage model had little explanatory power in total insect abundance.

PERMANOVA indicated that aquatic insect community composition was not significantly associated with 6PPD-Q concentration (*R* ^2^ = 0.021, *p* = 0.576; Figure S8A). There was also not a significant interaction between concentration × spike stage (*R* ^2^ = 0.013, *p* = 0.800). However, as community composition differed significantly between pre- and postspike sampling periods (*R* ^2^ = 0.062, *p* = 0.024), this suggests that temporal ecological turnover, rather than contaminant exposure, was the primary driver of compositional change. SIMPER analysis indicated that any pre- versus postspike differences in aquatic insect community composition were driven primarily by chironomid taxa and immature odanates. The subfamily chironominae (Family Chironomidae) increased in mean abundance following the spike period, whereas the subfamily tanypodinae (Family Chironomidae) increased. As well, there was a general decline in Chironomids not identified to the subfamily level. Immature odonates also increased postspike. Together, the four largest contributors accounted for approximately 74% of total Bray–Curtis dissimilarity. Diversity metrics were similarly not influenced by 6PPD-Q concentration, spike stage, or their interaction. Taxonomic richness showed no detectable treatment response (overall model *p* = 0.965; Figure S8B), and both Shannon (*p* = 0.399; Figure S8C) and Simpson diversity (*p* = 0.242; Figure S8D) similarly remained stable across treatments.

### 3.5 Effects on Embryo-Larval Fish

Exposure to 6PPD-Q significantly affected survival (ANOVA; *p* = 0.0058, *F*_5,20_ = 5.1924), with the 100 μg/L exposure group showing significantly lower survival compared to control (*p* = 0.0018) (Figure 2). Cumulative hatch rate significantly increased over the exposure period (*F*_1,177_ = 161.35; *p* < 0.0001), reflecting normal embryonic development across treatment groups. Here, dose did not significantly affect cumulative hatch rate (*F*_1,177_ = 12.16, *p* = 0.0601), but there was a significant interaction between dose and exposure day (*F*_5,177_ = 4.04, *p* = 0.0017), suggesting that hatch trajectory differed among doses over time. A Dunnett’s *post hoc* comparison showed that only the highest dose (100 μg/L) significantly differed from the control group (difference = −14.07; *t* = −2.97, adj. 95% CI: −26.24 to −1.90; Figure 3).

**Figure 2:**
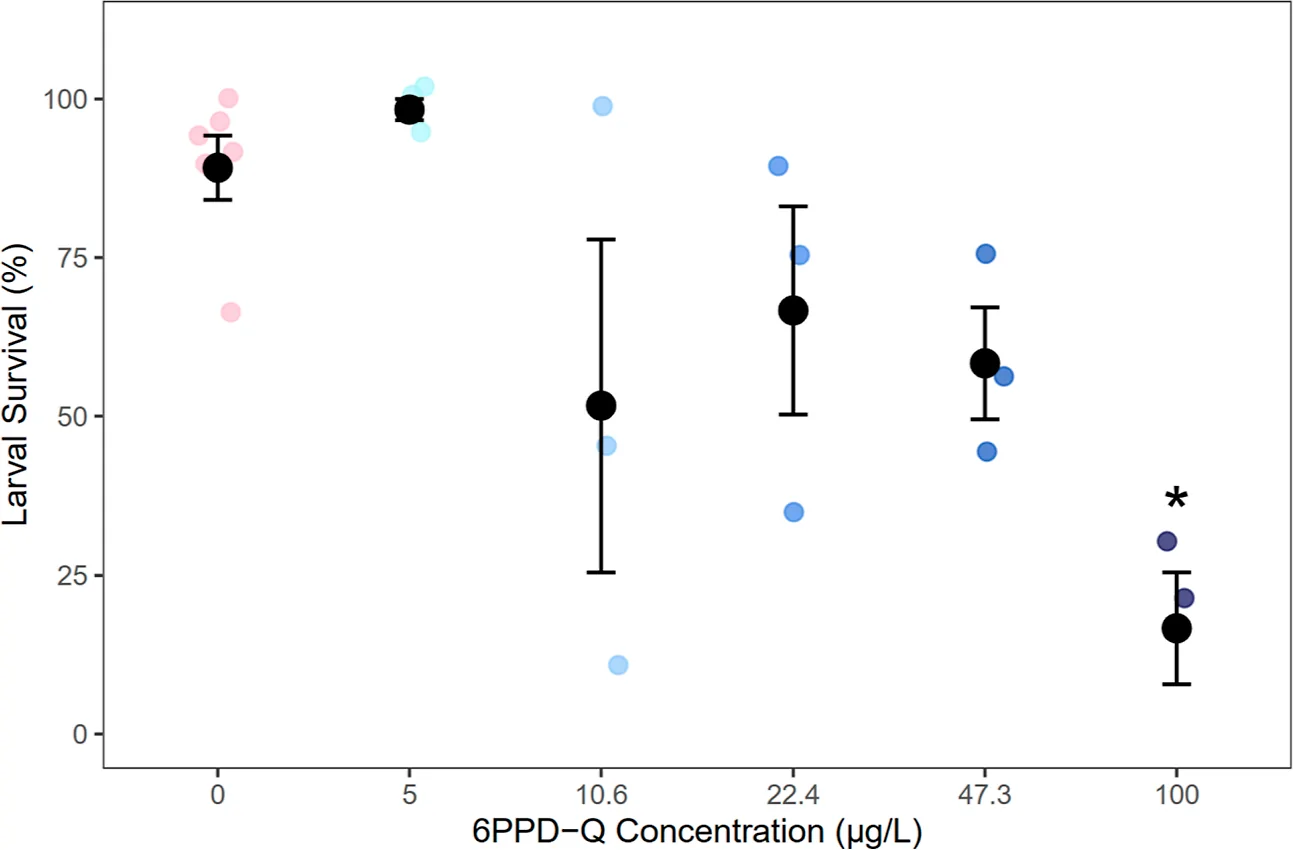
Larval survival of fathead minnow at day 8 following exposure to 6PPD-Q. Individual points represent survival in each replicate (*n* = 3/dose; *n* = 6 for control), with the mean ± standard error shown. Asterisk (*) indicates a significant difference from control. Statistical analyses are detailed in the text.

**Figure 3:**
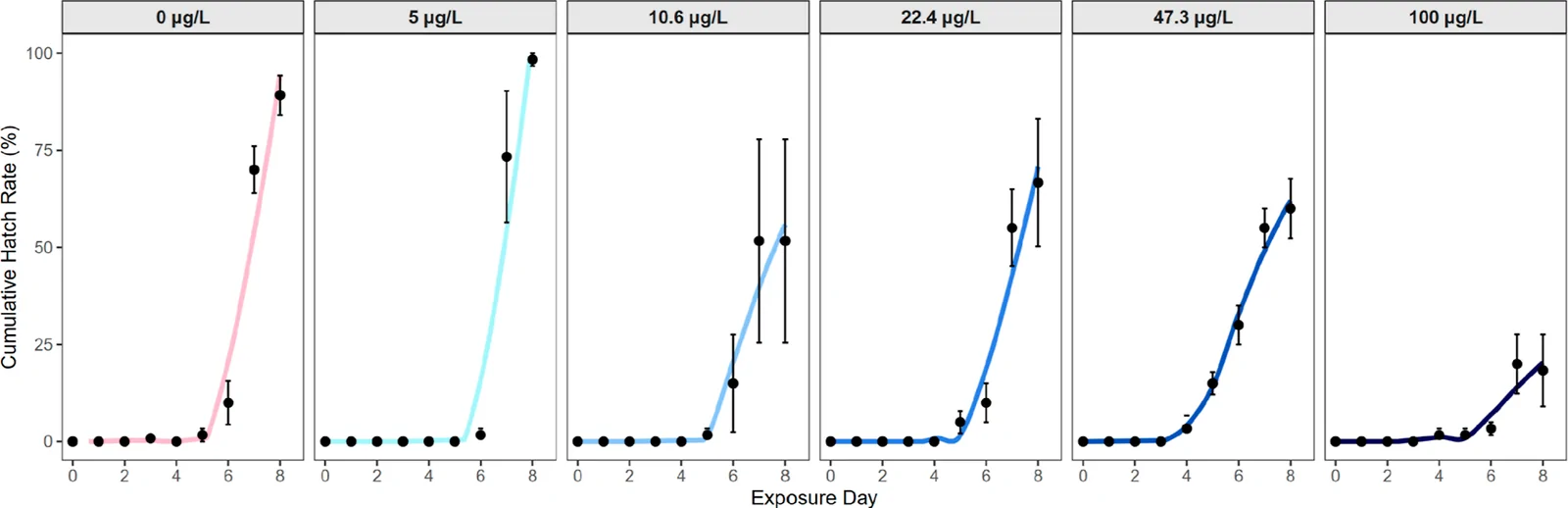
Cumulative hatch rate of fathead minnows over an 8-day exposure to 6PPD-Q. Each panel represents a dose group. Points represent mean ± standard error of replicate jars (*n* = 3/dose; *n* = 6 for control). The line represents the locally weighted smoothing function (LOESS). A significant dose x exposure day interaction was detected with the highest dose differing significantly from the control. Statistical analyses are detailed in the text.

Treatment had a significant effect on larval total length (*F*_5_ = 2.76, *p* = 0.05) and notochord length (*F*_5_ = 2.73, *p* = 0.054). *Post hoc* comparisons indicated that larvae exposed to the highest concentration of 6PPD-Q exhibited a ∼5.5% reduced total length (*p* = 0.023) and a ∼6% reduced notochord length (*p* = 0.015) relative to controls (Figure 4A). No significant treatment effects were observed for whole body area or relative body area (*p* > 0.05; Figure S9, Table 3). Similarly, no significant treatment-related differences were detected in brain area, pericardial area, swim bladder area, or relative brain, eye, and swim bladder areas (*p* > 0.05). In contrast, treatment had a significant effect on larval eye area (*F*_5_ = 2.871, *p* = 0.015; Figure 4B). *Post hoc* analysis revealed that larvae exposed to 10.6 μg/L exhibited an 11% significantly larger eye area compared to controls (*p* = 0.025); however, this effect was not observed for relative eye area (*p* > 0.05). A significant treatment effect was also observed for yolk sac area (*F*_5_ = 5.77, *p* = 4.4 × 10^–5^) and relative yolk sac area (*F*_5_ = 6.00, *p* = 2.74 × 10^–5^). *Post hoc* comparisons indicated that larvae exposed to 10.6 μg/L (*p* < 0.0001 for both end points) and 22.4 μg/L (*p* = 0.04 and *p* = 0.026, respectively) had significantly reduced yolk sac area and relative yolk sac area compared to controls (Figure 4C,E and Table 3). This resulted in a ∼24% and ∼8% smaller yolk sac area for the 10.6 and 22.4 μg/L, respectively; and a ∼22% and ∼11% smaller relative yolk sac area for the 10.6 and 22.4 μg/L, respectively. Finally, treatment significantly affected relative pericardial area (*F*_5_ = 5.79, *p* = 4.21 × 10^–5^). *Post hoc* comparisons showed that larvae exposed to 10.6 μg/L had a significantly smaller (∼40%) relative pericardial area (*p* = 0.014), whereas larvae exposed to 100 μg/L exhibited a significantly larger (∼40%) relative pericardial area (*p* = 0.0007) compared to controls (Figure 4D, Table 3). Visible differences in larval morphology among treatment concentrations are shown in Figure 5.

**Figure 4:**
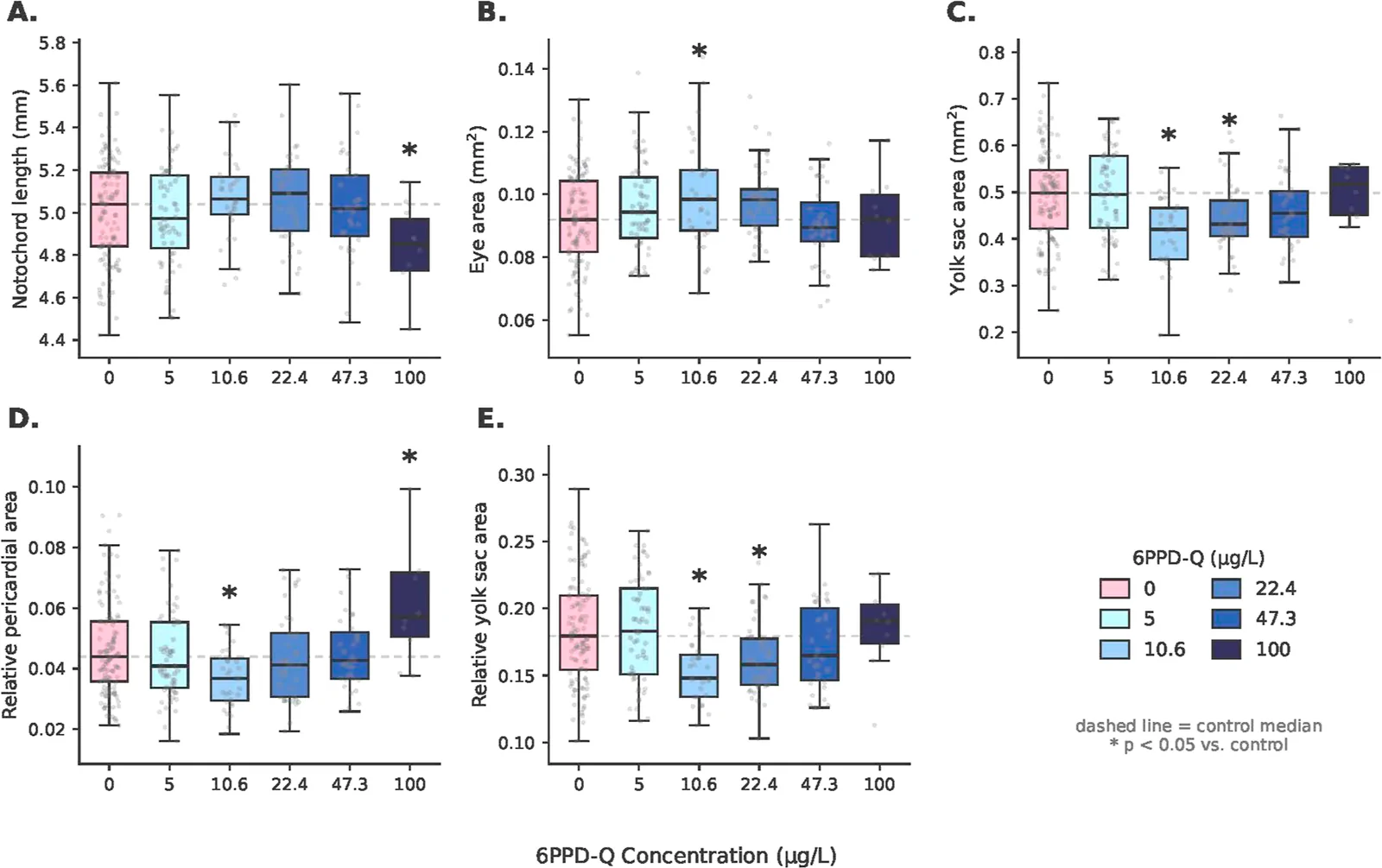
Larval fathead minnow morphometric end points showing significant treatment effects following an 8-day exposure to 6PPD-Q (0, 5, 10.6, 22.4, 47.3, and 100 μg/L). (A) Notochord length (mm), (B) eye area (mm^2^), (C) yolk sac area (mm^2^), (D) relative pericardial area, and (E) relative yolk sac area. Boxes represent the interquartile range (IQR) with the median indicated by the horizontal line; whiskers extend to the most extreme values within 1.5 × IQR. Individual larvae are overlaid as points, and the dashed line indicates the control median. An asterisk (*) indicates significance relative to controls (linear mixed effects model followed by Dunnett post hoc, α = 0.05). End points for which no significant treatment effect was detected are presented in Figure S9, and all morphometric data are tabulated in Table 3.

**Table 3:** Average (±Standard Deviation) Larval Fish Morphometric end Points Based on 6PPD-Q Exposure (5, 10.6, 22.4, 47.3, and 100 μg/L)^a^

		total length (mm)	notochord length (mm)	body area (mm^2^)	brain area (mm^2^)	pericardium (mm^2^)	eye area (mm^2^)	yolk sac area (mm^2^)	swim bladder area (mm^2^)	relative body area	relative brain area	relative pericardium	relative eye area	relative yolk sac area	relative swim bladder area
treatment	*n*	mean ± SD	mean ± SD	mean ± SD	mean ± SD	mean ± SD	mean ± SD	mean ± SD	mean ± SD	mean ± SD	mean ± SD	mean ± SD	mean ± SD	mean ± SD	mean ± SD
control	6	5.78 ± 0.13	5.02 ± 0.11	2.67 ± 0.09	0.04 ± 2.12 × 10^–3^	0.12 ± 8.46 × 10^–3^	0.09 ± 8.35 × 10^–3^	0.49 ± 0.04	0.06 ± 0.02	461 ± 8.35	0.02 ± 1.52 × 10^–3^	0.05 ± 5.27 × 10^–3^	0.04 ± 9.81 × 10^–3^	0.19 ± 0.02	0.02 ± 0.01
5 μg/L	3	5.73 ± 0.10	4.99 ± 0.10	2.70 ± 0.03	0.04 ± 2.16 × 10^–3^	0.12 ± 2.86 × 10^–2^	0.10 ± 3.61 × 10^–3^	0.50 ± 0.05	0.06 ± 0.03	470 ± 7.63	0.02 ± 9.38 × 10^–4^	0.04 ± 0.01	0.04 ± 1.28 × 10^–3^	0.18 ± 0.02	0.02 ± 9.61 × 10^–3^
10.6 μg/L	3	5.93 ± 0.09*	5.07 ± 0.05	2.72 ± 0.04	0.05 ± 0.01	0.10 ± 0.01*	0.10 ± 8.09 × 10^–3^*	0.35 ± 0.05*	0.08 ± 0.02*	461 ± 4.25	0.02 ± 3.21 × 10^–3^	0.03 ± 4.03 × 10^–3^	0.04 ± 2.55 × 10^–3^	0.14 ± 0.02	0.03 ± 7.86 × 10^–3^*
22.4 μg/L	3	5.82 ± 0.09	5.08 ± 0.04	2.74 ± 0.05	0.05 ± 2.83 × 10^–3^	0.12 ± 7.39 × 10^–3^	0.10 ± 5.93 × 10^–3^	0.45 ± 0.02	0.06 ± 0.01	471 ± 1.44	0.02 ± 7.37 × 10^–4^	0.04 ± 2.73 × 10^–3^	0.04 ± 1.88 × 10^–3^	0.16 ± 0.01	0.02 ± 3.2 × 10^–3^
47.3 μg/L	3	5.78 ± 0.01	5.03 ± 0.03	2.69 ± 0.04	0.04 ± 3.05 × 10^–3^	0.13 ± 1.58 × 10^–2^	0.09 ± 5.23 × 10^–3^	0.45 ± 0.05	0.06 ± 0.01	466 ± 6.75	0.01 ± 1.39 × 10^–3^	0.05 ± 5.64 × 10^–3^	0.033 ± 2.13 × 10^–3^	0.17 ± 0.02	0.02 ± 5.05 × 10^–3^
100 μg/L	2	5.60 ± 0.21	4.84 ± 0.22	2.56 ± 0.07	0.04 ± 3.22 × 10^–4^	0.16 ± 1.55 × 10^–2^*	0.09 ± 1.49 × 10^–2^	0.49 ± 0.03	0.02 ± 0.01	469 ± 11.2	0.02 ± 4.3 × 10^–3^	0.07 ± 5.6 × 10^–3^	0.04 ± 5.3 × 10^–3^	0.19 ± 0.02	0.01 ± 2.27 × 10^–3^

a An asterisk (*) indicates significance relative to controls (linear mixed effect model followed by Dunnett *post hoc*, α = 0.05). Relative body area = body area relative to fish total length (area/length); relative brain area = brain relative to fish body area (brain/body area); relative pericardium = pericardium relative to fish body area (pericardium/body area); relative eye area = eye relative to fish body area (eye/body area); relative yolk sac area = yolk sac relative to fish body area (yolk sac/body area); relative swim bladder area = swim bladder relative to fish body area (swim bladder/body area).

**Figure 5:**
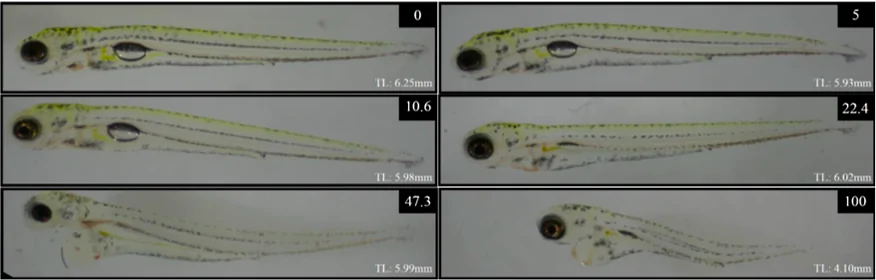
Representative images of fathead minnow larvae exposed to increasing 6PPD-Q concentrations. Larvae are shown from control 0 μg/L and treatment groups (5, 10.6, 22.4, 47.3, and 100 μg/L), with total length (TL) indicated for each representative individual. Notable morphological changes are apparent at high concentrations, including reduced length and developmental abnormalities.

## 4 Discussion

6PPD-Q has emerged as a globally pervasive contaminant of aquatic ecosystems. Monitoring studies conducted across North America and other regions have consistently detected 6PPD-Q in surface runoff, urban streams, wastewater, stormwater, and sediments, frequently at concentrations exceeding acute toxicity thresholds for sensitive aquatic species.^35^ Although toxicity data are available for 25 aquatic species, with eight identified as particularly sensitive, the current body of evidence remains insufficient to fully characterize the ecological risk posed by 6PPD-Q.^35^

### 4.1 Analytical Verification of 6PPD-Q, Biofilm, Photosynthetic Potential, and Phytoplankton Community Composition

Because the nominal concentrations in the higher treatments exceeded the reported aqueous solubility of 6PPD-Q, precipitation and subsequent redissolution cannot be excluded as factors influencing the measured concentrations. In addition, partitioning to suspended particles, sediments, and biota, together with degradation, likely influenced the fate of 6PPD-Q within the mesocosms. Therefore, the observed persistence of 6PPD-Q should be interpreted cautiously, as multiple physicochemical and biological processes likely contributed to the measured concentration patterns.

While biofilm development was strongly influenced by time, there was no detectable response to 6PPD-Q exposure. Chlorophyll-*a* concentrations were substantially higher when sampled postspike, indicating pronounced accumulation of photosynthetic biomass over the course of the experiment. Chlorophyll-*a* was not significantly associated with treatment concentration, which suggests that natural biofilm colonization and growth processes drove algal biomass dynamics, as opposed to 6PPD-Q.^23,36^ As mesocosm experiments progress there are often substantial increases in pigment biomass through time because of increasing habitat stability.^37^ An absence of a treatment effect indicates that any toxicological influence of 6PPD-Q on algal accrual was either minimal, transient, or masked by the magnitude of natural growth processes occurring during the study period.

Likewise, AFDM, which reflects total organic biofilm biomass, also showed no relationship with treatment concentration; overall organic matter accumulation remained stable among exposures, further supporting the conclusion that 6PPD-Q did not substantially alter biofilm biomass production. When interpreted alongside the chlorophyll-*a* results, these data suggest that both autotrophic and total biofilm mass developed similarly among treatments. In mesocosm systems, AFDM often includes algal biomass, heterotrophic microbes, extracellular matrix material, and trapped detritus as benthic biofilms mature through time, even in the absence of treatment effects.^23,36^

Phytoplankton communities were unaffected by 6PPD-Q concentration across all response variables examined. Total biomass, total density, taxa richness, Shannon diversity, Simpson diversity, and multivariate community composition showed no significant effects because of 6PPD-Q concentration. There were also no significant interaction effects between concentration and spike stage for any of the tested response variables. Together, these results suggest that, under the conditions tested, 6PPD-Q did not measurably alter phytoplankton standing stocks, biodiversity, or assemblage structure across the concentration range evaluated.

### 4.2 Zooplankton Sex Ratios, Age Ratios, Total Abundance, and Community Structure

6PPD-Q was not found to influence the total abundances, sex ratios, age ratios, or community compositions of zooplankton; any variations in abundance were more strongly associated with the sampling date. The sex ratio (measured as the proportion of females to males) remained relatively constant across treatments and sampling periods. Neither linear modeling nor rank-based correlation analyses indicated any significant associations between dose and sex ratio, and no temporal trends were observed. As sex ratios are used in ecological data to predict implications toward reproductive output and long-term community persistence, results would indicate that there were no sex specific sensitivities or long-term implications.^38,39^ While total zooplankton abundance varied significantly across sampling dates, sex ratios remained stable. This would indicate that temporal fluctuations in population size were not accompanied by shifts in population structure. Adult life-stage proportions similarly remained stable across treatments and sampling periods, suggesting no significant disruption of developmental progression under the tested concentrations. There was a weak trend toward elevated nauplii abundance at midstudy sampling, but this was not statistically nor biologically significant and was likely a reflection of natural temporal cohort turnover.^24,40^

NMDS and PERMANOVA analyses indicated that zooplankton assemblage structures were primarily influenced by sampling date rather than by the concentration of 6PPD-Q. Community composition shifted significantly over the course of the experiment, but this was the temporal succession that is commonly observed in enclosed mesocosm systems. This occurs as communities respond to changing food availability, reproduction pulses, competitive interactions, and other naturally occurring dynamics.^24,40^ Treatment concentration alone did not significantly explain community-level variation, although a marginal association could be observed when sampling date was included in the model. This suggests that any potential treatment-related responses were subtle relative to the stronger background signal of natural temporal turnover.

Patterns in alpha diversity further support this interpretation. Taxonomic richness increased significantly by the final sampling date, while Shannon and Simpson diversity indices showed a reduction during the middle date of the study, which was followed by increased evenness at the final sampling period. This trend could be a result of short-term dominance by a subset of opportunistic and/or competitive taxa, followed by rediversification as the community matures.^26,40^ Collectively, these results suggest that the zooplankton communities in this study were not significant impacted by the tested concentrations of 6PPD-Q, and that natural successional processes were the dominant drivers of any observed variation. Exposure did not produce strong or consistent disruptions to abundance, demographic structure, or overall community organization.

### 4.3 Benthic Insect Total Abundance and Community Structure

Benthic insect communities were largely resilient to 6PPD-Q exposure under the experimental conditions tested. Across all univariate end points examined (total abundance, taxonomic richness, Shannon diversity, and Simpson diversity), there were no detectable effects of 6PPD-Q dose. There were also no significant interaction effects between 6PPD-Q dose and the spike stage, which would indicate that the community structure of benthic insects did not significantly differ across time or because of exposure. Temporal dynamics appeared to have been the only driving force behind community abundance and structure and were found to be statistically significant when analyzing multivariate community composition. PERMANOVA found a significant shift between pre- and postspike assemblages, while treatment concentration itself remained nonsignificant. This would indicate that natural ecological turnover was likely the primary driver of community change during the study period.^41^ This could be due to seasonal succession; aquatic insect communities often undergo rapid shifts in relative abundance as cohorts transition among life stages.^41,42^ Any changes that we saw to proportional representation of our insect orders (Ephemeroptera, Odonata, Trichoptera, and Diptera) was gradual overtime, rather than being either abrupt or dependent on the concentration of 6PPD-Q (Figure S7).

SIMPER analysis further indicated that pre- versus postspike differences were driven primarily by chironomids (Diptera: Chironomidae) and immature Odonata. Chironomids are well-known for rapid generation times and opportunistic colonization of habitats (including Hester-Dendys).^42,43^ The subfamily chironominae increased following the spike period, whereas tanypodinae and unidentified chironomid groups declined. Immature odonates became more abundant as the study progressed. As previously stated, these patterns are consistent with normal successional turnover rather than contaminant-induced influence on sensitive taxa.

### 4.4 Embryo-Larval Fathead Minnow

Significant effects on survival and hatch dynamics were observed only at the highest exposure concentration (100 μg/L), which greatly exceeds concentrations reported in both surface waters and episodic stormwater runoff, where 6PPD-Q typically occurs in the ng/L to low μg/L range.^4,35^ Accordingly, these responses represent an extremely high-exposure scenario rather than environmentally realistic conditions. However, the observed sensitivity of early life stages at elevated concentrations indicates the potential for adverse effects under extreme runoff events. While such outcomes are unlikely under typical ambient exposures, the possibility of sublethal or transient effects at environmentally relevant concentrations remains and warrants further investigation.

Although morphological responses were most pronounced at higher exposure levels, several statistically significant effects were also detected at intermediate concentrations (10.6 and 22.4 μg/L), including reductions in total yolk sac area and relative yolk sac area, as well as altered pericardial and eye morphologies. This aligns with work in larval zebrafish, which were also found to have reduced eye sizes and impacted cardiac performance at similar 6PPD-Q concentrations (10 and 25 μg/L), potentially suggesting similar impacts on development among freshwater species.^44^ These concentrations, however, exceed those so far measured in the environment. The revised quantification of Tian et al.^2^ (which reanalyzed archived extracts against a commercial analytical standard) lowered previously reported environmental concentrations of 6PPD-Q by approximately an order of magnitude. Applying that correction, the highest credibly reported concentrations in stormwater and surface waters remain in the low μg/L range (approximately 2.8 μg/L) in an urban Canadian watershed^5^ and <3.0 μg/L in West Coast roadway runoff.^2^ The effects observed here at 10.6 and 22.4 μg/L therefore occurred roughly an order of magnitude above current environmental measurements, and are best interpreted as hazard characterization rather than as evidence of risk under present ambient conditions.

Previous work found no significant impacts of 6PPD-Q on survival, hatch success, or development of lab-reared fathead minnows up to 39.27 μg/L, suggesting that wild-caught fathead minnow embryos are comparatively more sensitive to 6PPD-Q.^12^ The magnitude and consistency of yolk sac depletion and cardiac-related morphological changes seen here suggest that early developmental processes may be sensitive to transient exposure peaks, even if such concentrations are episodic in nature. Smaller yolk sac reserves may be a negative consequence in early life stage fish in the wild as larger energy stores correspond with faster growth rates, leading to better odds of survival.^45,46^ Further, decreased relative pericardial size can lead to impaired structural development and limited cardiac capacity, while increased relative pericardial size can be indicative of cardiac edema.^47^ Even subtle cardiac defects during early life stages can lead to long-lasting reductions in growth, swimming ability, and survival, which translate directly into lower recruitment and population-level fitness impacts.^48^ Together, these results identify yolk sac depletion and altered pericardial morphology as sensitive developmental end points for 6PPD-Q in this species, albeit at concentrations above those currently documented in urban watersheds. Should such concentrations be approached during extreme first-flush events, or should sensitivity prove greater under exposures longer or more frequent than the 7-day window tested here, alterations of this kind during critical early life stages could translate into reduced larval performance, compromised energetic reserves, and ultimately decreased survival and recruitment potential at the population level. Further, differences in fish source (wild versus laboratory-reared), genetic background, and prior environmental history may contribute to variation in sensitivity; however, these factors were not directly evaluated in the present study and therefore cannot be conclusively assessed.

While early investigations of 6PPD-Q demonstrated pronounced sensitivity among salmonid species-with rapid mortality in a subset of species following environmentally relevant exposures—subsequent studies have also identified a broader spectrum of sublethal effects in nonsalmonid species.^6–8,12,35,44,49^ For example, fathead minnow exposure has been associated with increased oxidative stress, while zebrafish exhibits reduced heart rate and altered behavioral responses.^12,44^ An important direction for future research would be direct comparisons between wild and laboratory-reared fathead minnows, as well as long-term assessments of growth, behavior, swimming performance, and reproduction following embryonic exposure, would provide valuable insight into the mechanisms underlying potential differences in sensitivity.

Overall, this study demonstrates that while 6PPD-Q did not measurably alter lower trophic levels, including biofilm, phytoplankton, zooplankton, and benthic insect communities, under the tested conditions, ELS fish exhibited both lethal and sublethal responses at concentrations above those presently reported in the environment. These findings establish developmental hazard thresholds for a nonsalmonid species and indicate that any ecological risk associated with 6PPD-Q would be most pronounced during episodic high-exposure events, where transient concentration spikes may impair fish development and recruitment, highlighting the need for further investigation into pulsed exposure dynamics and long-term population-level consequences in urban-impacted watersheds.

## Supplementary Material



## Notes

Daily mesocosm temperature, dissolved oxygen and light intensity data; larval morphology end point diagram; biofilm chlorophyll-*a* and ash-free dry mass; phytoplankton community composition and diversity; zooplankton community composition, population structure and alpha diversity; aquatic insect community composition by order; benthic insect community composition and diversity; larval morphometric end points without significant treatment effects (PDF)

## Abbreviations

6PPD-quinone (6PPD-Q): 2-((4-methylpentan-2-yl)­amino)-5-(phenylamino)­cyclohexa-2,5-diene-1,4-dione
AFDM: Ash free dry mass
ANOVA: analysis of variance
D5-6PPD-Q: deuterated 6PPD-Q
DIC: dissolved inorganic carbon
DL: desolvation line
DMSO: dimethyl sulfoxide
DOC: dissolved organic carbon
ELS: early life stage
Fe: suspended iron
HPLC: high-performance liquid chromatography
HPLC/MS: triple-quadruple mass spectrometer
IACUC: Institutional Animal Care and Use Committee
IISD-ELA: International Institute for Sustainable Development—Experimental Lakes Area
LC_50_: lethal Concentration 50
LCMS: liquid chromatography–mass spectrometry
LOEC: lowest observed effect concentration
MRM: multireaction monitoring
NMDS: nonmetric multidimensional scaling
P: suspended phosphorus
PERMNOVA: permutational multivariate analysis of variance
Si: reactive silica
SIMPER: similarity percentages analysis
SO_4_: sulfate
TDN: total dissolved nitrogen

## References

[1] Ackerly K. L., Roark K. J., Lu K., Esbaugh A. J., Liu Z., Nielsen K. M. (2024). Acute toxicity testing of 6PPD-quinone on the estuarine-dependent
sport fish, Sciaenops ocellatus. Ecotoxicology.

[2] Tian Z., Gonzalez M., Rideout C. A., Zhao H. N., Hu X., Wetzel J., Mudrock E., James C. A., McIntyre J. K., Kolodziej E. P. (2022). 6PPD-Quinone:
Revised Toxicity Assessment and Quantification
with a Commercial Standard. Environ. Sci. Technol.
Lett..

[3] Tian Z., Zhao H., Peter K. T., Gonzalez M., Wetzel J., Wu C., Hu X., Prat J., Mudrock E., Hettinger R., Cortina A. E., Biswas R. G., Kock F. V. C., Soong R., Jenne A., Du B., Hou F., He H., Lundeen R., Gilbreath A., Sutton R., Scholz N. L., Davis J. W., Dodd M. C., Simpson A., McIntyre J. K., Kolodziej E. P. (2021). A ubiquitous tire rubber-derived chemical induces acute
mortality in coho salmon. Science.

[4] Helm P. A., Raby M., Kleywegt S., Sorichetti R. J., Arabian G., Smith D., Howell E. T., Thibeau J. (2024). Assessment
of Tire-Additive Transformation Product 6PPD-Quinone in Urban-Impacted
Watersheds. ACS EST Water.

[5] Johannessen C., Helm P., Lashuk B., Yargeau V., Metcalfe C. D. (2022). The Tire
Wear Compounds 6PPD-Quinone and 1,3-Diphenylguanidine in an Urban
Watershed. Arch. Environ. Contam. Toxicol..

[6] Shankar P., Dalsky E. M., Salzer J. E., Lane R. F., Hammond S., Batts W. N., Gregg J. L., Greer J. B., Kurath G., Hershberger P. K., Hansen J. D. (2025). Evaluation of 6PPD-Quinone Lethal
Toxicity and Sublethal Effects on Disease Resistance and Swimming
Performance in Coastal Cutthroat Trout (Oncorhynchus clarkii clarkii). Environ. Sci. Technol..

[7] Hiki K., Yamamoto H. (2022). The Tire-Derived Chemical 6PPD-quinone Is Lethally
Toxic to the White-Spotted Char Salvelinus leucomaenis pluvius but
Not to Two Other Salmonid Species. Environ.
Sci. Technol. Lett..

[8] Brinkmann M., Montgomery D., Selinger S., Miller J. G. P., Stock E., Alcaraz A. J., Challis J. K., Weber L., Janz D., Hecker M., Wiseman S. (2022). Acute Toxicity of the
Tire Rubber-Derived
Chemical 6PPD-quinone to Four Fishes of Commercial, Cultural, and
Ecological Importance. Environ. Sci. Technol.
Lett..

[9] Roberts C., Lin J., Kohlman E., Jain N., Amekor M., Alcaraz A. J., Hogan N., Hecker M., Brinkmann M. (2025). Acute and
Subchronic Toxicity of 6PPD-Quinone to Early Life Stage Lake Trout
(Salvelinus namaycush). Environ. Sci. Technol..

[10] Foldvik A., Kryuchkov F., Sandodden R., Uhlig S. (2022). Acute Toxicity Testing
of the Tire Rubber-Derived Chemical 6PPD-quinone on Atlantic Salmon
(Salmo salar) and Brown Trout (Salmo trutta). Environ. Toxicol. Chem..

[11] Hiki K., Asahina K., Kato K., Yamagishi T., Omagari R., Iwasaki Y., Watanabe H., Yamamoto H. (2021). Acute Toxicity
of a Tire Rubber-Derived Chemical, 6PPD Quinone, to Freshwater Fish
and Crustacean Species. Environ. Sci. Technol.
Lett..

[12] Anderson-Bain K., Roberts C., Kohlman E., Ji X., Alcaraz A. J., Miller J., Gangur-Powell T., Weber L., Janz D., Hecker M., Montina T., Brinkmann M., Wiseman S. (2023). Apical and mechanistic effects of
6PPD-quinone on different
life-stages of the fathead minnow (Pimephales promelas). Comp. Biochem. Physiol., Part C: Toxicol. Pharmacol..

[13] Prosser R. S., Salole J., Hang S. (2023). Toxicity of 6PPD-quinone
to four
freshwater invertebrate species. Environ. Pollut..

[14] Brunskill G. J., Schindler D. W. (1971). Geography and Bathymetry of Selected
Lake Basins, Experimental
Lakes Area, Northwestern Ontario. J. Fish. Res.
Board Can..

[15] Santschi P. H., Nyffeler U. P., Anderson R. F., Schiff S. L., O’Hara P., Hesslein R. H. (1986). Response of Radioactive
Trace Metals to Acid-Base Titrations
in Controlled Experimental Ecosystems: Evaluation of Transport Parameters
for Application to Whole-Lake Radiotracer Experiments. Can. J. Fish. Aquat. Sci..

[16] Levine S. N., Schindler D. W. (1999). Influence of Nitrogen to Phosphorus Supply Ratios and
Physicochemical Conditions on Cyanobacteria and Phytoplankton Species
Composition in the Experimental Lakes Area, Canada. Can. J. Fish. Aquat. Sci..

[17] Christensen M. R., Graham M. D., Vinebrooke R. D., Findlay D. L., Paterson M. J., Turner M. A. (2006). Multiple Anthropogenic Stressors Cause Ecological Surprises
in Boreal Lakes. Global Change Biol..

[18] Tank S. E., Xenopoulos M. A., Hendzel L. L. (2005). Effect of Ultraviolet Radiation on
Alkaline Phosphatase Activity and Planktonic Phosphorus Acquisition
in Canadian Boreal Shield Lakes. Limnol. Oceanogr..

[19] Vinebrooke R. D., Turner M. A., Kidd K. A., Hann B. J., Schindler D. W. (2001). Truncated
Foodweb Effects of Omnivorous Minnows in a Recovering Acidified Lake. J. North Am. Benthol. Soc..

[50] Havens, S. M. , Cooney, B. T. , Stainton, M. P. The chemical analysis of fresh water. 2024, 3rd ed. International Institute for Sustainable Development - Experimental Lakes Area (IISD-ELA). https://www.iisd.org/publications/guide/chemical-analysis-fresh-water-third-edition

[20] Blandford N. C., Palace V., Rodríguez-Gil J. L., Timlick L., Stanley M., Frank R. A., Campbell S., Rudy M., Marshall S., Wynia A., Clark T., Cunningham J., Alaee M., Parrott J., Kidd K. A., Stevack K., Sabourin L., Renaud J. B., Sumarah M. W., Nielsen K., McMaster M. E., Ussery E. (2024). Community-Level Responses
and Environmental
Fate of Metformin in Freshwater Mesocosms. Sci.
Total Environ..

[21] Nielsen K. M., DeCamp L., Birgisson M., Palace V. P., Kidd K. A., Parrott J. L., McMaster M. E., Alaee M., Blandford N., Ussery E. J. (2022). Comparative Effects
of Embryonic Metformin Exposure
on Wild and Laboratory-Spawned Fathead Minnow (Pimephales promelas)
Populations. Environ. Sci. Technol..

[22] Stevenson, R. J. An Introduction to Algal Ecology in Freshwater Benthic Habitats. Algal Ecology: Freshwater Benthic Ecosystems; Stevenson, R. J. , Bothwell, M. L. , Lowe, R. L. , Eds.; Academic Press: San Diego, CA, 1996; pp 3–30. 10.1016/B978-012668450-6/50030-8.

[23] Hauer, F. R. ; Lamberti, G. A. , Eds. Methods in Stream Ecology; Ecosystem Structure, 3rd ed.; Academic Press: London, 2017; Vol. 1.

[51] R Core Team . R: A language and environment for statistical computing (Version 4.4), 2024; R Foundation for Statistical Computing.https://www.R-project.org/

[24] Wetzel, R. G. Limnology: Lake and River Ecosystems, 3rd ed.; Academic Press: San Diego, CA, 2001.

[25] Reynolds, C. S. The Ecology of Phytoplankton; Ecology, Biodiversity and Conservation; Cambridge University Press: Cambridge, U.K., 2006. 10.1017/CBO9780511542145.

[26] Magurran, A. E. Measuring Biological Diversity; Blackwell Publishing: Oxford, U.K., 2004.

[27] Bray J. R., Curtis J. T. (1957). An Ordination of the Upland Forest
Communities of Southern
Wisconsin. Ecol. Monogr..

[28] Kruskal J. B. (1964). Nonmetric
Multidimensional Scaling: A Numerical Method. Psychometrika.

[29] Anderson M. J. (2001). A New Method
for Non-Parametric Multivariate Analysis of Variance. Austral Ecol..

[30] Clarke, K. R. ; Warwick, R. M. Change in Marine Communities: An Approach to Statistical Analysis and Interpretation, 2nd ed.; PRIMER-E: Plymouth, U.K., 2001.

[31] Clarke K. R. (1993). Non-Parametric
Multivariate Analyses of Changes in Community Structure. Aust. J. Ecol..

[32] Spearman C. (1904). The Proof
and Measurement of Association between Two Things. Am. J. Psychol..

[33] Oksanen, J. ; Simpson, G. L. ; Blanchet, F. G. ; Kindt, R. ; Legendre, P. ; Minchin, P. R. ; O’Hara, R. B. ; Solymos, P. ; Stevens, M. H. H. ; Szoecs, E. ; Wagner, H. ; vegan: Community Ecology Package, R package version 2.6-4, 2024. https://CRAN.R-project.org/package=vegan (accessed April 15, 2026).

[34] Merritt, R. W. , Cummins, K. W. , Eds.; An Introduction to the Aquatic Insects of North America, 3rd ed.; Kendall/Hunt: Dubuque, IA, 1996.

[35] Wang S., Xu S., Lu J., Wan Y., Kang Z., Chen H., Islam S., Gao B. (2026). Occurrence, Transformation, and Toxicity
of Tire-Derived Chemicals 6PPD and 6PPD-q in the Environment. Environ. Sci. Technol..

[36] Lock, M. A. River Epilithon: A Light and Organic Energy Transducer. Perspectives in Running Water Ecology; Lock, M. A. , Williams, D. D. , Eds.; Plenum Press: New York, 1981; pp 3–40. 10.1007/978-1-4684-1122-5_2.

[37] Battin T. J., Kaplan L. A., Denis Newbold J., Hansen C. M. E. (2003). Contributions
of Microbial Biofilms to Ecosystem Processes in Stream Mesocosms. Nature.

[38] Hazlerigg C. R. E., Tyler C. R., Lorenzen K., Wheeler J. R., Thorbek P. (2014). Population
Relevance of Toxicant Mediated Changes in Sex Ratio in Fish: An Assessment
Using an Individual-Based Zebrafish (Danio rerio) Model. Ecol. Modell..

[39] Wedekind C. (2017). Demographic
and Genetic Consequences of Disturbed Sex Determination. Philos. Trans. R. Soc., B.

[40] Lampert, W. ; Sommer, U. Limnoecology: The Ecology of Lakes and Streams, 2 ed.; Haney, J. F. , Ed.; Oxford University Press: Oxford, U.K., 2007.

[41] Magurran, A. E. , McGill, B. J. , Eds.; Biological Diversity: Frontiers in Measurement and Assessment; Oxford University Press: Oxford, 2011.

[42] Allan, J. D. ; Castillo, M. M. Stream Ecology: Structure and Function of Running Waters, 2nd ed.; Springer: Dordrecht, 2007. 10.1007/978-1-4020-5583-6.

[43] Armitage, P. D. , Cranston, P. S. , Pinder, L. C. V. , Eds. The Chironomidae: The Biology and Ecology of Non-biting Midges; Chapman & Hall: London, 1995. 10.1007/978-94-011-0715-0.

[44] Varshney S., Gora A. H., Siriyappagouder P., Kiron V., Olsvik P. A. (2022). Toxicological
Effects of 6PPD and 6PPD Quinone in Zebrafish Larvae. J. Hazard. Mater..

[45] Garrido S., Ben-Hamadou R., Santos A. M. P., Ferreira S., Teodósio M. A., Cotano U., Irigoien X., Peck M. A., Saiz E., Ré P. (2015). Born Small, Die Young: Intrinsic, Size-Selective Mortality
in Marine Larval Fish. Sci. Rep..

[46] Sulaeman, Fotedar R. (2017). Yolk Utilization and
Growth during the Early Larval Life of the Silver Perch, Bidyanus
bidyanus (Mitchell, 1838). Int. Aquat. Res..

[47] Incardona J. P., Scholz N. L. (2016). The Influence of
Heart Developmental Anatomy on Cardiotoxicity-Based
Adverse Outcome Pathways in Fish. Aquat. Toxicol..

[48] Incardona J. P., Carls M. G., Holland L., Linbo T. L., Baldwin D. H., Myers M. S., Peck K. A., Tagal M., Rice S. D., Scholz N. L. (2015). Very Low Embryonic
Crude Oil Exposures Cause Lasting
Cardiac Defects in Salmon and Herring. Sci.
Rep..

[49] Philibert D., Stanton R. S., Tang C., Stock N. L., Benfey T., Pirrung M., de Jourdan B. (2024). The Lethal
and Sublethal Impacts
of Two Tire Rubber-Derived Chemicals on Brook Trout (Salvelinus fontinalis)
Fry and Fingerlings. Chemosphere.

