# Supplementary material for: Evaluating Community-Level Effects of 6PPD-Quinone in a Natural Boreal Lake Ecosystem
Source: Environ Sci Technol. 2026 Oct 16;60(43):30427–39. doi: 10.1021/acs.est.6c08085 (PMC13738693; doi:10.1021/acs.est.6c08085)
Supplement: Supplementary file 1 [file es6c08085_si_001.pdf]

Evaluating community-level effects of 6PPD-quinone in a natural boreal lake ecosystem

Erin J Ussery [a*], Kerri Lynn Ackerly [b], Kathleen J Roark [b], Jose Luis Rodriguez-Gil [c], Lauren Timlick [c], Vince Palace [c], Alon R. Agua [d], Michael Pirrung [d], Kristin Nielsen [b]

[a] Environment and Climate Change Canada, Burlington, Ontario, L7S 1A1, Canada

[b] The University of Texas at Austin, Department of Marine Science, Port Aransas, TX, 78373. USA

[c] International Institute for Sustainable Development - Experimental Lakes Area, Winnipeg, Manitoba, R3B 0X7, Canada

[d] University of California Riverside, Department of Chemistry, Riverside, CA, 92521, USA

*Corresponding Author: Erin Ussery; Erin.Ussery@ec.gc.ca

Number of Pages: 14

Number of Figures: 9

Number of Tables: 1

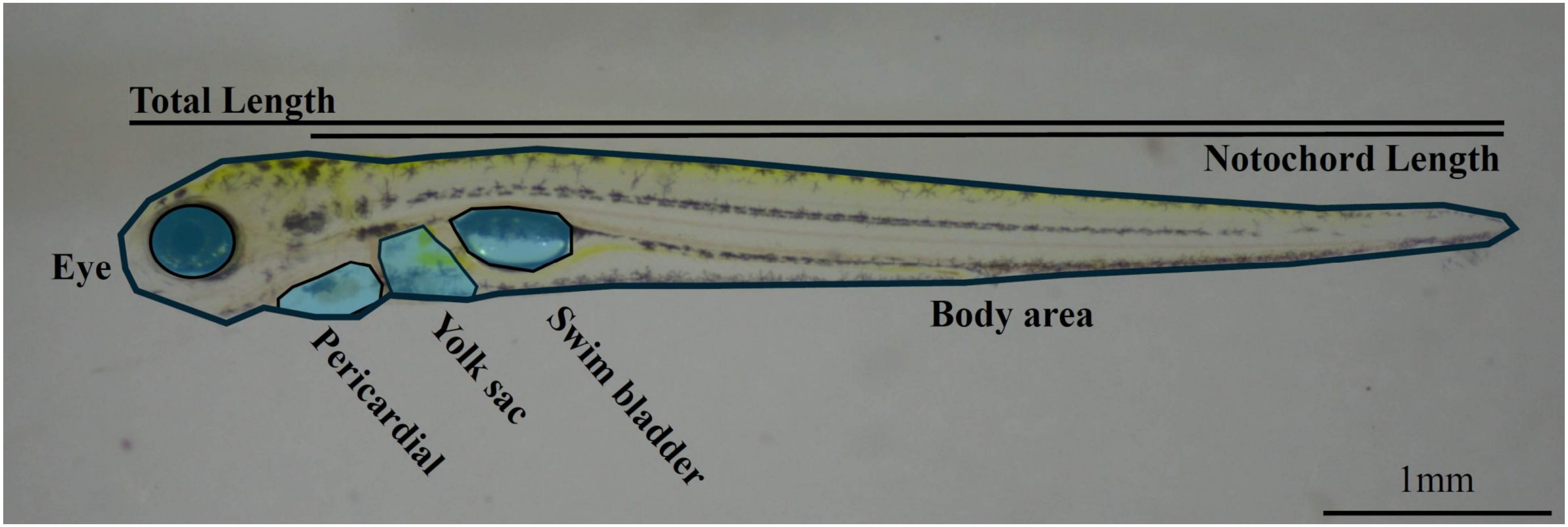

Figure S1. Morphological endpoints measured in larval fathead minnows. Photograph of a representative larva with annotated regions of interest used for image analysis, including total length, notochord length, total body area, eye area, pericardial area, yolk sac area, and swim bladder area measured using image analysis software.

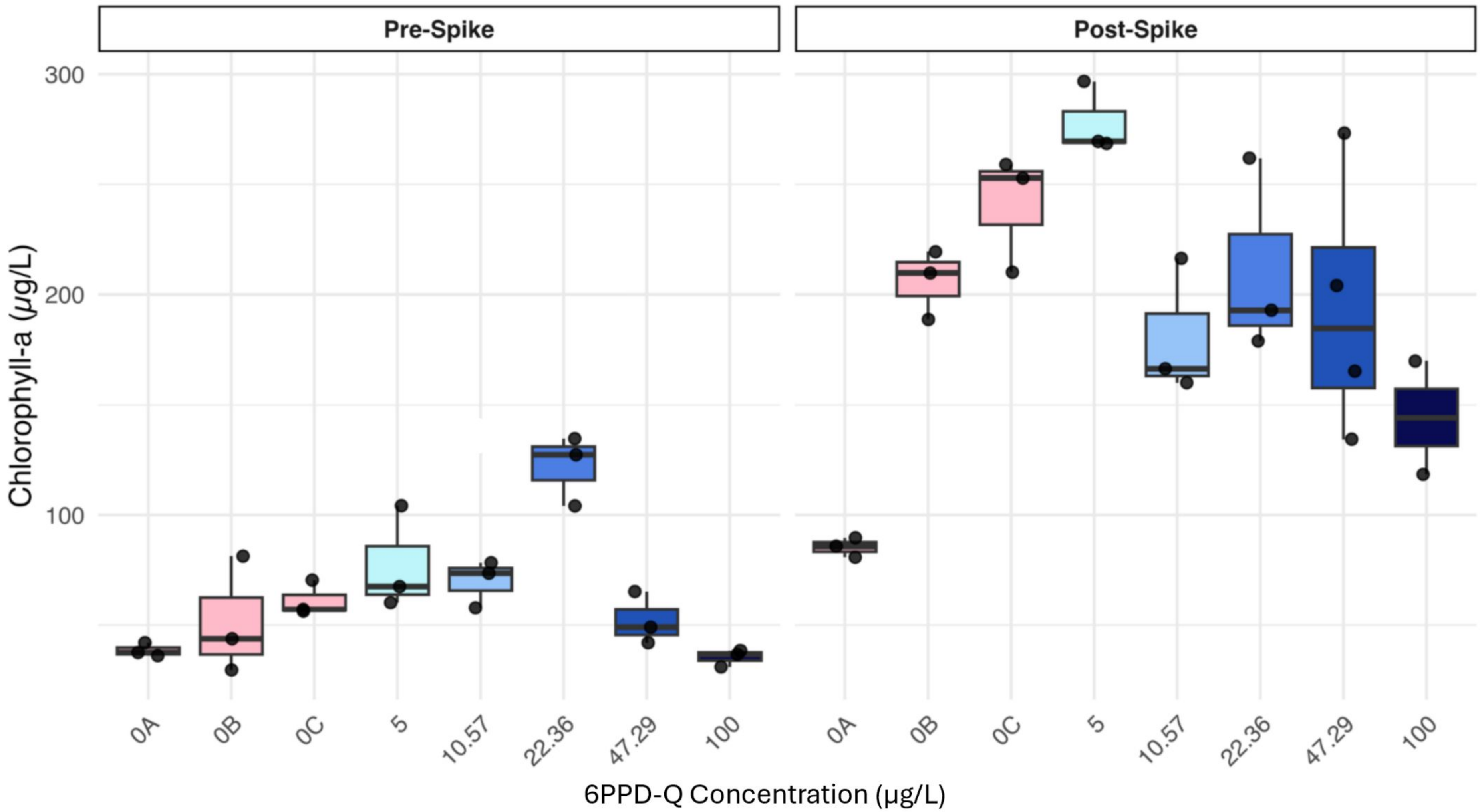

Figure S2. Chlorophyll-a concentrations (μg/L) across 6PPD-Q treatment concentrations before (pre-spike) and after (post-spike) compound addition. Boxplots represent the distribution of measurements within each treatment, with points representing individual observations. The center line represents the median, the boxes represent the interquartile range (IQR), and whiskers represent the most extreme values within 1.5 x IQR.

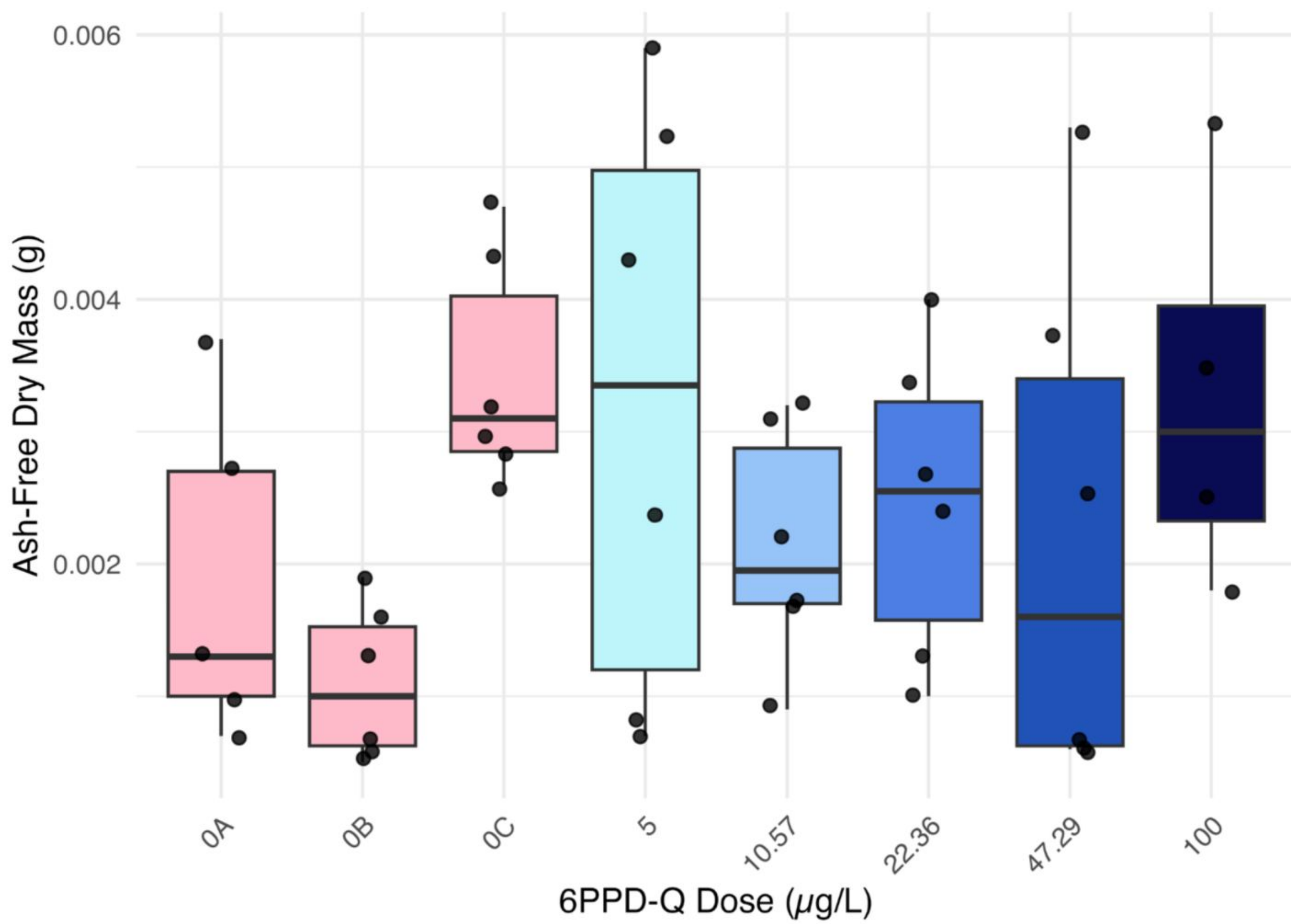

Figure S3. Ash-free dry mass (AFDM; g) across 6PPD-Q treatment concentrations. Boxplots represent the distribution of replicate measurements within each treatment, where the center line indicates the median, boxes denote the interquartile range (IQR), whiskers extend to the most extreme values within 1.5 × IQR, and points represent individual observations.

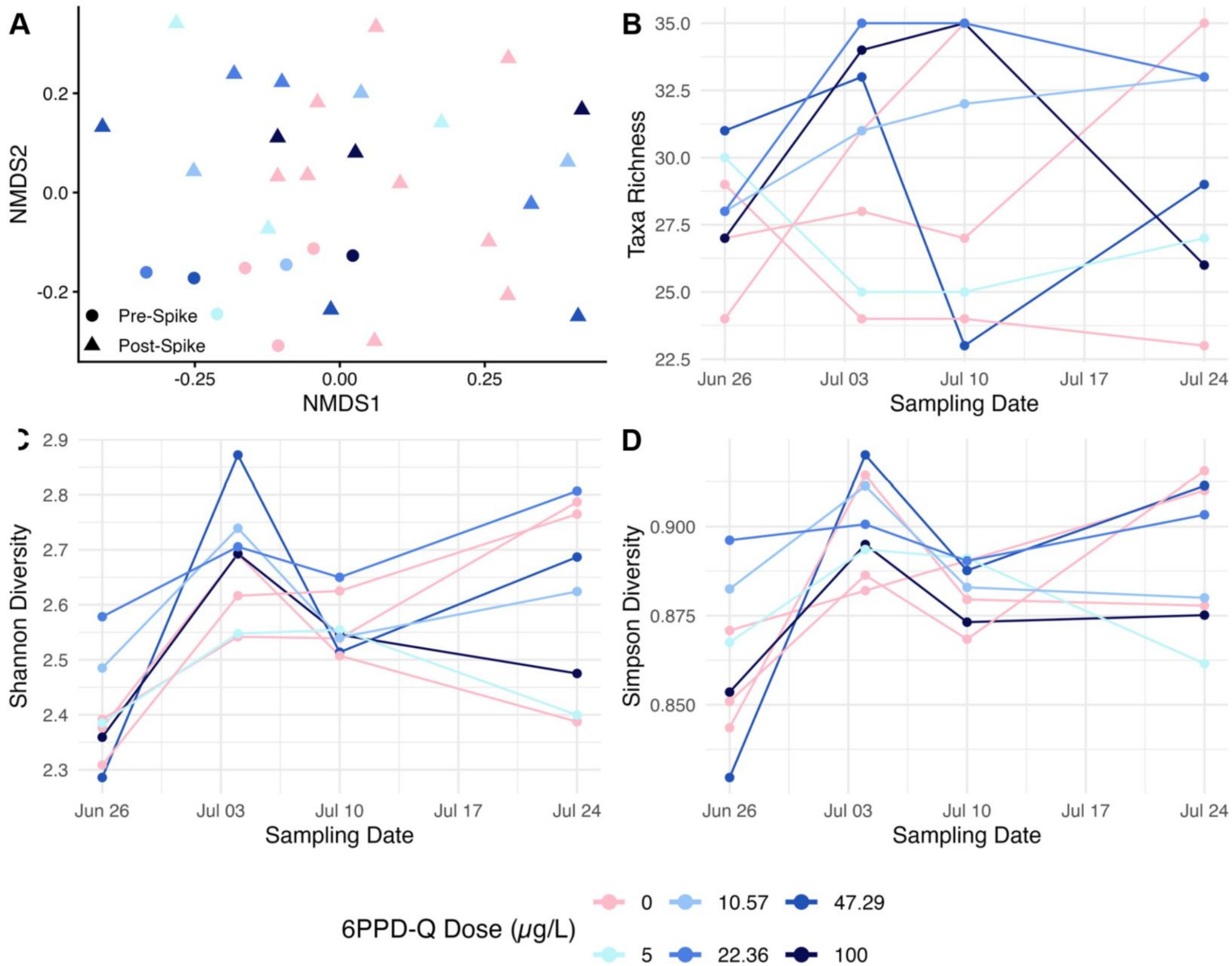

Figure S4. Phytoplankton community composition and diversity across sampling dates and 6PPD-Q treatment concentrations. (A) Non-metric multidimensional scaling (NMDS) ordination of phytoplankton community composition based on Bray–Curtis dissimilarities, with symbols indicating pre-spike and post-spike samples. (B) Taxa richness, (C) Shannon diversity, and (D) Simpson diversity are shown across sampling dates for each treatment. Lines connect repeated measurements within treatments, and points represent individual mesocosm observations.

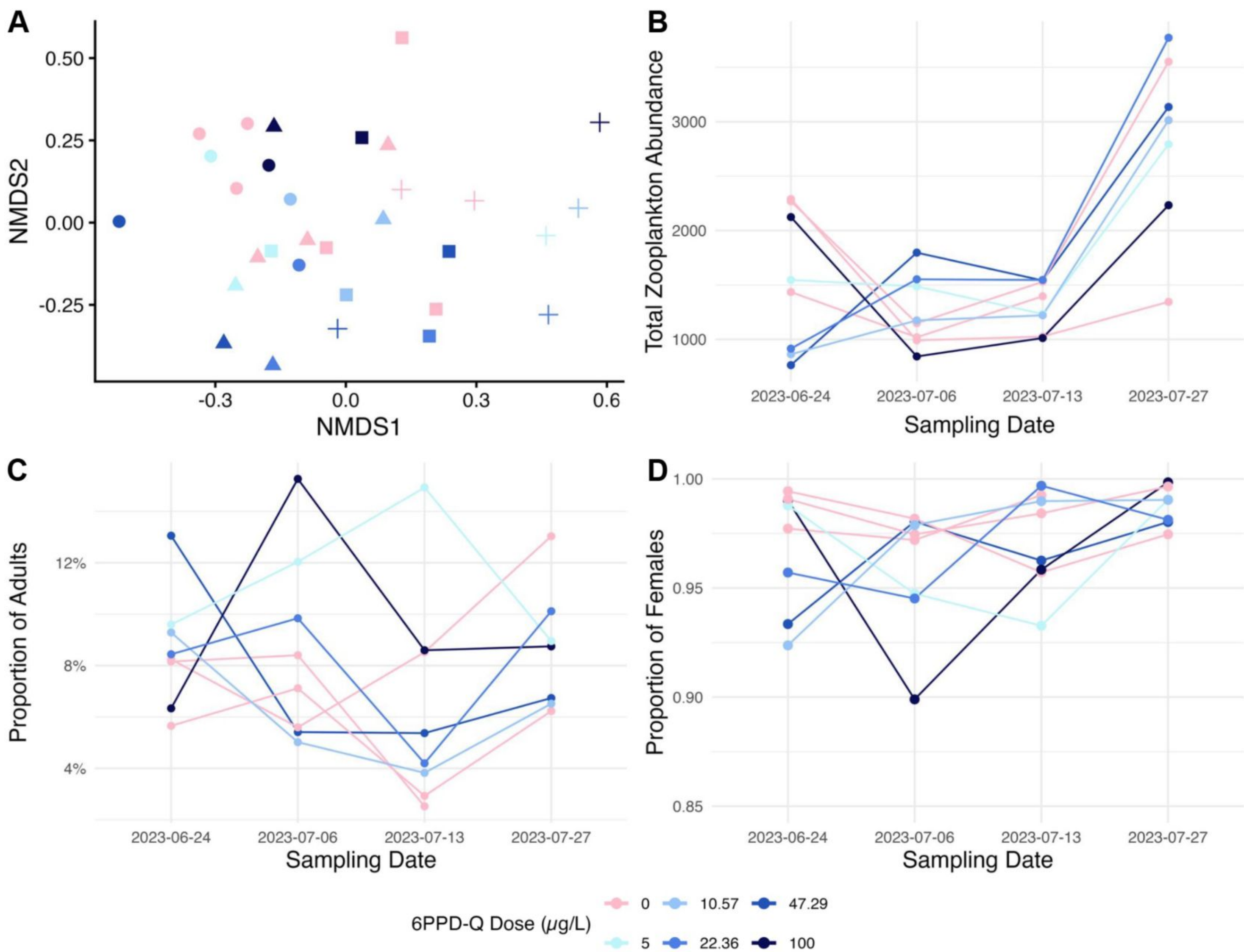

Figure S5. Zooplankton community composition and population structure across sampling dates and 6PPD-Q treatment concentrations. (A) Non-metric multidimensional scaling (NMDS) ordination of community composition based on Bray–Curtis dissimilarities. (B) Total zooplankton abundance, (C) proportion of adults, and (D) proportion of females across sampling dates for each treatment. Lines connect repeated measurements within treatments, and points represent individual mesocosm observations.

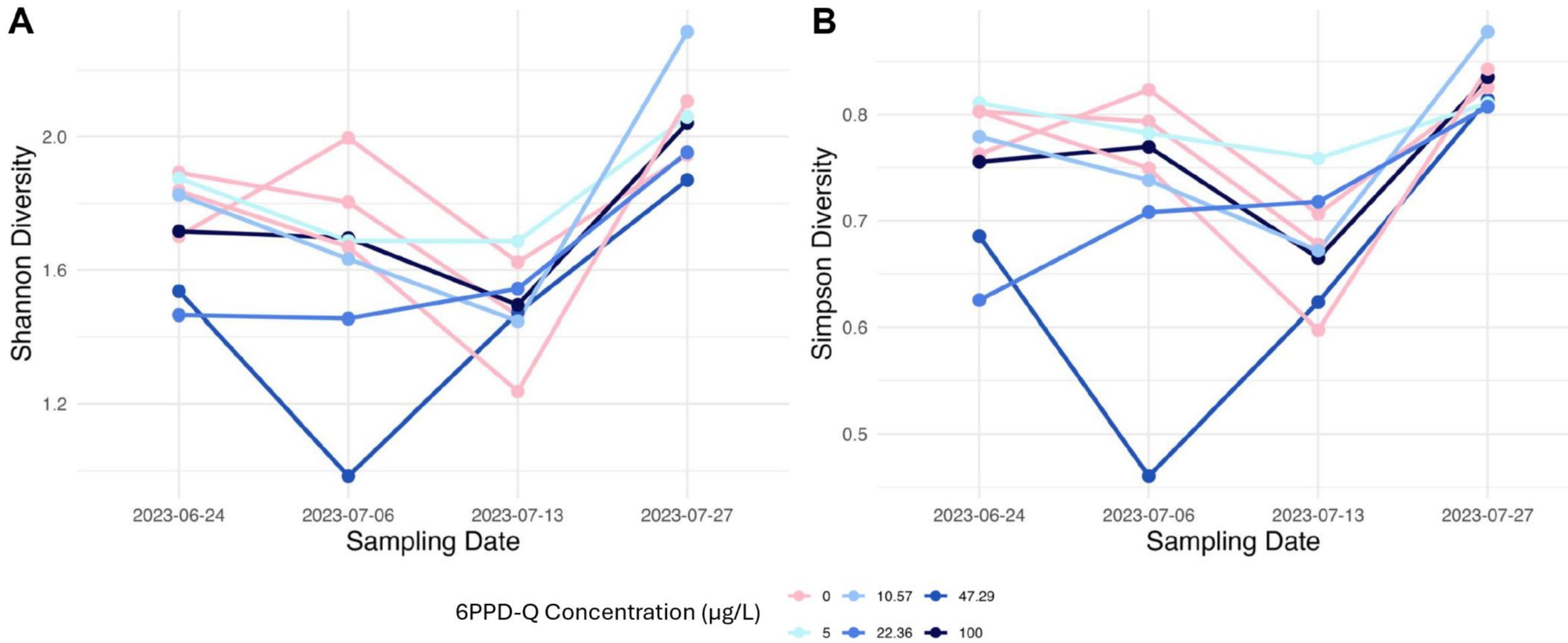

Figure S6. Zooplankton alpha diversity across sampling dates (x-axis) and 6PPD-Q treatment concentrations (individual lines). (A) Shannon diversity and (B) Simpson diversity are displayed for each treatment overtime. Points represent individual mesocosm observations.

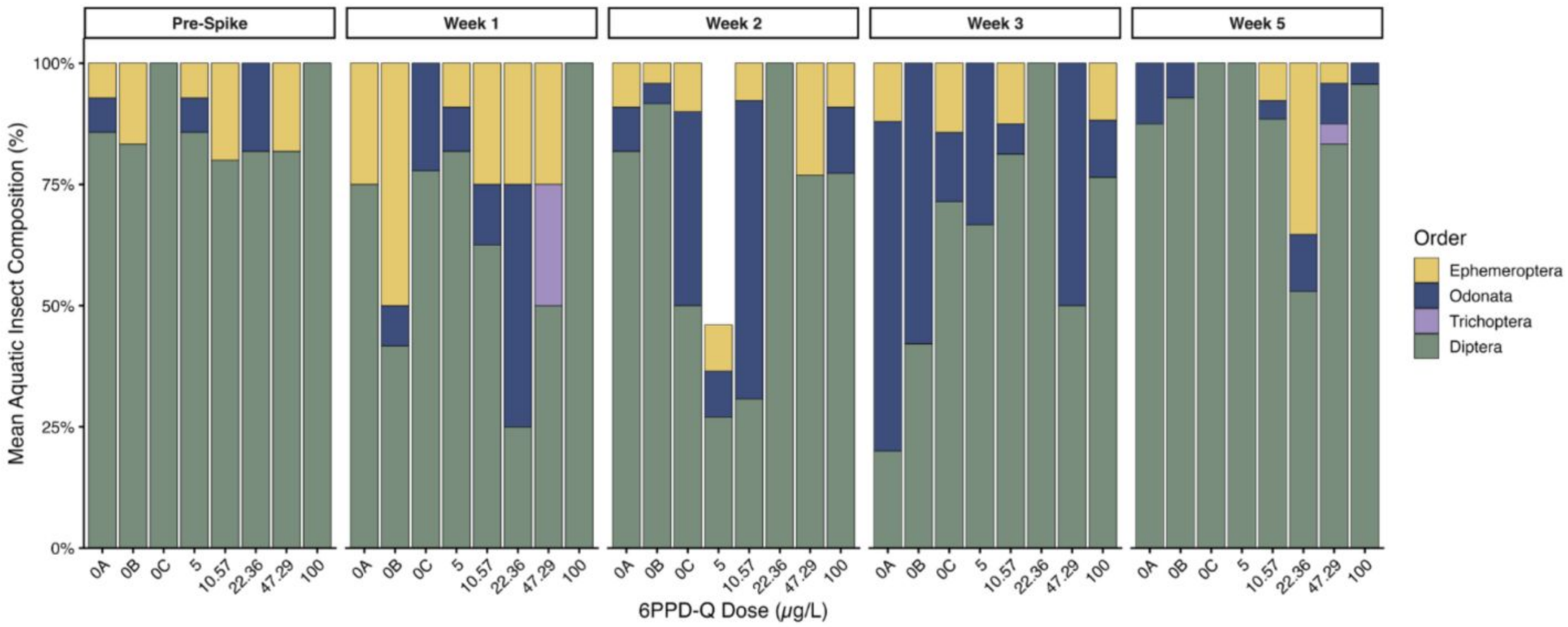

Figure S7. Mean aquatic insect community composition (%) by order across 6PPD-Q treatment concentrations and sampling periods. Bars represent the proportional contribution of each taxonomic order (Ephemeroptera, Odonata, Trichoptera, and Diptera) within each treatment at each sampling period (Pre-Spike, Week 1, Week 2, Week 3, and Week 5). Values are averaged across replicate samples within each treatment and sampling period.

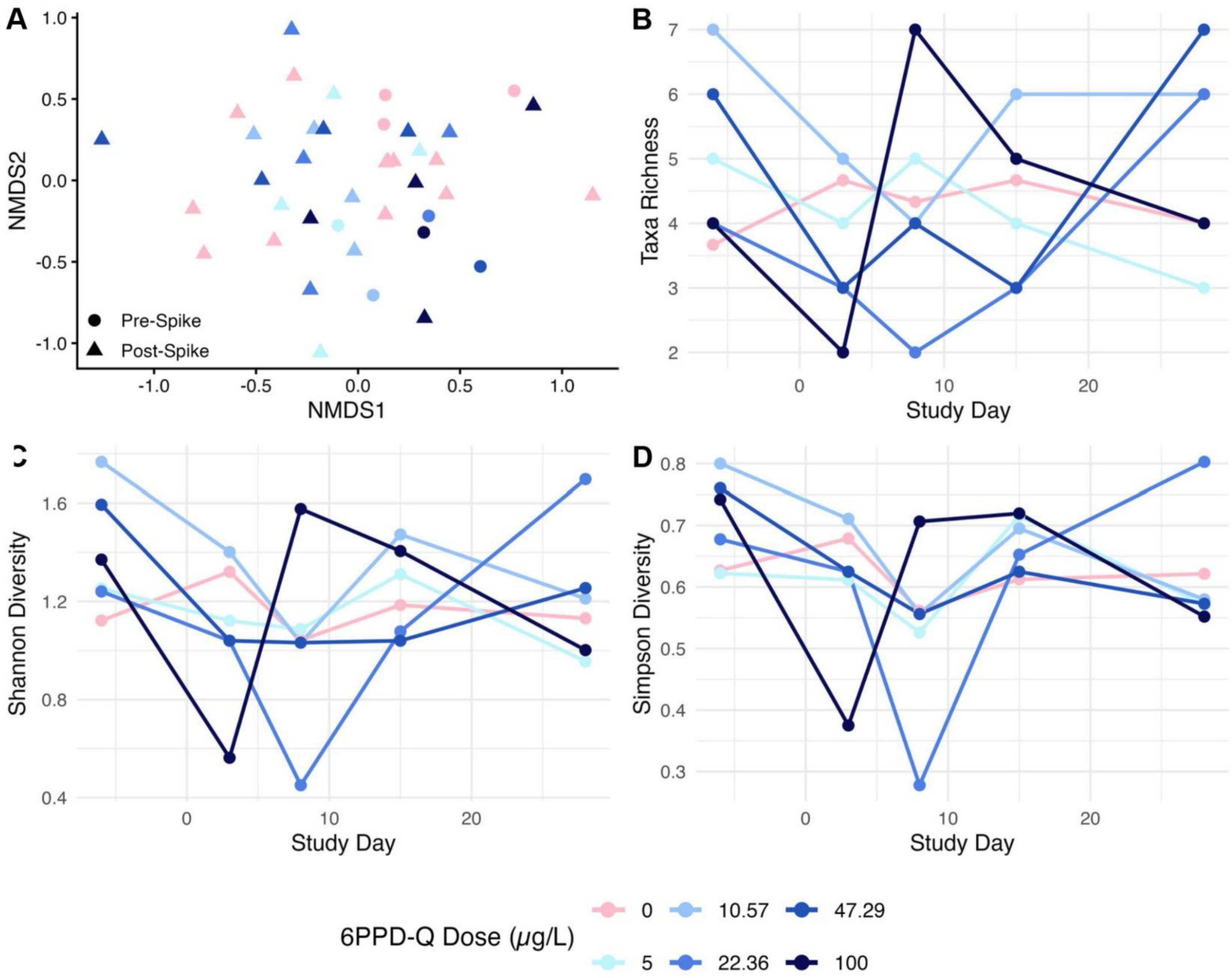

Figure S8. Benthic insect community composition and diversity across sampling dates and 6PPD-Q treatment concentrations. (A) Non-metric multidimensional scaling (NMDS) ordination of macroinvertebrate community composition based on Bray–Curtis dissimilarities, with symbols indicating pre-spike and post-spike samples. (B) Taxa richness, (C) Shannon diversity, and (D) Simpson diversity are shown across sampling dates for each treatment. Lines connect repeated measurements within treatments, and points represent individual mesocosm observations.

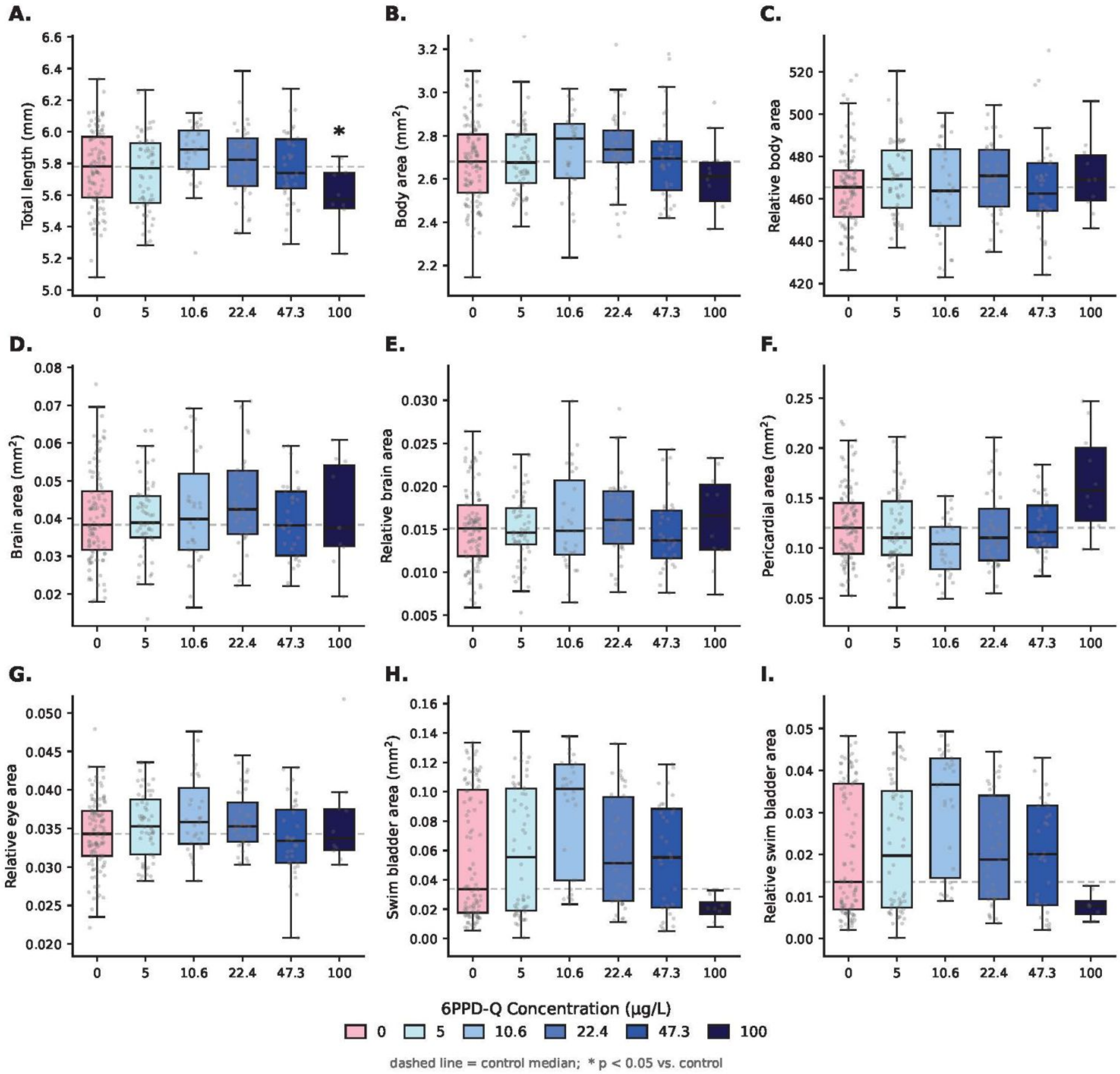

Figure S9. Larval fathead minnow morphometric endpoints for which no significant treatment effect was detected, following an 8-day exposure to 6PPD-Q (0, 5, 10.6, 22.4, 47.3 and 100 µg/L). (A) Total length (mm), (B) body area (mm2), (C) relative body area, (D) brain area (mm2), (E) relative brain area, (F) pericardial area (mm2), (G) relative eye area, (H) swim bladder area (mm2), and (I) relative swim bladder area. Boxes represent the interquartile range (IQR) with the median indicated by the horizontal line; whiskers extend to the most extreme values within 1.5 x IQR. Individual larvae are overlaid as points, and the dashed line indicates the control median. An asterisk (*) indicates significance relative to controls (linear mixed effects model followed by Dunnett post hoc, $\alpha = 0.05$). Total length is presented here because it is redundant with notochord length, which is presented in Figure 4.

Table S1.

Mesocosm	Date	Temperature (°C)	Dissolved Oxygen (mg/L)	Light (Lux)
1	7/14/2023	21.2 ± 0.02	8.55 ± 0.02	6746.58 ± 389.45
1	7/15/2023	20.5 ± 0.01	8.54 ± 0.01	6156.64 ± 307.32
1	7/16/2023	19.99 ± 0.02	8.53 ± 0.01	5415.17 ± 309.39
1	7/17/2023	19.79 ± 0.01	8.58 ± 0	4680.65 ± 259.69
1	7/18/2023	20 ± 0.03	8.55 ± 0.01	7574.01 ± 356.91
1	7/19/2023	20.15 ± 0.02	8.62 ± 0.01	4569.77 ± 252.35
1	7/20/2023	20.77 ± 0.05	8.59 ± 0.01	9019.83 ± 446.11
1	7/21/2023	21.27 ± 0.02	8.67 ± 0.01	8630.95 ± 498.22
1	7/22/2023	21.75 ± 0.05	8.66 ± 0	8541.88 ± 403.05
1	7/23/2023	21.64 ± 0.02	8.71 ± 0.01	6799.08 ± 293.94
1	7/24/2023	21.76 ± 0.04	8.65 ± 0.01	11087.38 ± 556.95
1	7/25/2023	21.62 ± 0.01	8.62 ± 0	5155.25 ± 269.49
1	7/26/2023	22.33 ± 0.05	8.52 ± 0	9857.09 ± 473.81
1	7/27/2023	22.88 ± 0.03	8.47 ± 0	9630.76 ± 480.82
1	7/28/2023	22.23 ± 0.02	8.4 ± 0.01	4927.6 ± 227.45
1	7/29/2023	21.61 ± 0.02	8.4 ± 0.01	8090.03 ± 432.72
1	7/30/2023	21.68 ± 0.03	8.44 ± 0.01	8099.43 ± 364
1	7/31/2023	21.66 ± 0.02	8.42 ± 0	5202.37 ± 237.84
2	7/14/2023	21.25 ± 0.02	7.77 ± 0.01	7353.05 ± 430.78
2	7/15/2023	20.53 ± 0.01	7.62 ± 0	6593.52 ± 340.82
2	7/16/2023	20.04 ± 0.02	7.69 ± 0.01	6438.76 ± 381.96
2	7/17/2023	19.84 ± 0.01	7.72 ± 0	5219.82 ± 298.28
2	7/18/2023	20.06 ± 0.03	7.69 ± 0.01	8463.95 ± 406.3
2	7/19/2023	20.17 ± 0.02	7.76 ± 0.01	4898.15 ± 273.95
2	7/20/2023	20.81 ± 0.05	7.8 ± 0.01	10141.81 ± 572.62
2	7/21/2023	21.29 ± 0.02	7.86 ± 0.01	6823.42 ± 384.8
2	7/22/2023	21.81 ± 0.05	7.75 ± 0.01	8699.22 ± 420.91
2	7/23/2023	21.67 ± 0.02	7.85 ± 0.01	7962.58 ± 358.35
2	7/24/2023	21.78 ± 0.04	7.79 ± 0.01	11044.49 ± 560.49
2	7/25/2023	21.63 ± 0.01	7.77 ± 0	4462.94 ± 223.09
2	7/26/2023	22.38 ± 0.05	7.62 ± 0.01	12140.44 ± 597.49
2	7/27/2023	22.92 ± 0.03	7.69 ± 0.01	11789.73 ± 590.78
2	7/28/2023	22.25 ± 0.02	7.64 ± 0.01	5692.05 ± 266.8
2	7/29/2023	21.67 ± 0.02	7.71 ± 0.01	8577.33 ± 470.72
2	7/30/2023	21.72 ± 0.03	7.86 ± 0.01	7958.71 ± 388.55
2	7/31/2023	21.68 ± 0.02	7.95 ± 0.01	5281.97 ± 242.46
3	7/14/2023	21.23 ± 0.02	8.68 ± 0.02	7742.87 ± 498.06
3	7/15/2023	20.54 ± 0.01	8.69 ± 0.01	5718.87 ± 303.21
3	7/16/2023	20.03 ± 0.02	8.68 ± 0.01	5028.59 ± 305.02
3	7/17/2023	19.84 ± 0.01	8.66 ± 0	4064.91 ± 243.39

3	7/18/2023	20.04 ± 0.03	8.63 ± 0.01	7583.44 ± 388.74
3	7/19/2023	20.17 ± 0.02	8.7 ± 0.01	4572.86 ± 289.42
3	7/20/2023	20.81 ± 0.05	8.64 ± 0	12668.39 ± 682.03
3	7/21/2023	21.29 ± 0.02	8.72 ± 0	9626.62 ± 554.99
3	7/22/2023	21.81 ± 0.05	8.64 ± 0.01	11665.17 ± 615.73
3	7/23/2023	21.67 ± 0.02	8.69 ± 0.01	7261.05 ± 344.56
3	7/24/2023	21.78 ± 0.04	8.62 ± 0.01	14248.3 ± 746.18
3	7/25/2023	21.64 ± 0.01	8.61 ± 0	4680.79 ± 258.48
3	7/26/2023	22.4 ± 0.05	8.46 ± 0.01	13567.55 ± 699.64
3	7/27/2023	22.9 ± 0.03	8.42 ± 0.01	10745.7 ± 539.01
3	7/28/2023	22.27 ± 0.02	8.26 ± 0.01	4723.96 ± 224.3
3	7/29/2023	21.68 ± 0.02	8.25 ± 0.01	9596.29 ± 559.65
3	7/30/2023	21.74 ± 0.03	8.25 ± 0.01	10490.39 ± 527.23
3	7/31/2023	21.71 ± 0.02	8.27 ± 0.01	4958.02 ± 233.98
4	7/14/2023	21.44 ± 0.04	8.31 ± 0.01	6875.09 ± 472.18
4	7/15/2023	20.61 ± 0.01	8.24 ± 0.01	5669.28 ± 344.07
4	7/16/2023	20.09 ± 0.02	8.19 ± 0.01	6875.73 ± 519.72
4	7/17/2023	19.9 ± 0.01	8.22 ± 0.01	5089.94 ± 312.33
4	7/18/2023	20.09 ± 0.03	7.98 ± 0.01	8385.13 ± 421.79
4	7/19/2023	20.23 ± 0.01	8.05 ± 0.01	5656.89 ± 377.16
4	7/20/2023	20.88 ± 0.05	8.08 ± 0.01	13372.02 ± 754.27
4	7/21/2023	21.35 ± 0.02	7.78 ± 0.02	9157.83 ± 546.28
4	7/22/2023	21.85 ± 0.05	7.71 ± 0.02	10645.98 ± 544.65
4	7/23/2023	21.72 ± 0.02	7.86 ± 0.01	7701.72 ± 354.7
4	7/24/2023	21.81 ± 0.04	7.68 ± 0.02	15007.5 ± 794.66
4	7/25/2023	21.69 ± 0.01	7.81 ± 0.02	4220.11 ± 229.36
4	7/26/2023	22.43 ± 0.05	7.85 ± 0.02	13283.2 ± 702.19
4	7/27/2023	22.96 ± 0.03	8.06 ± 0.01	10713.08 ± 633.92
4	7/28/2023	22.33 ± 0.02	7.99 ± 0.01	5325.4 ± 272.19
4	7/29/2023	21.71 ± 0.02	7.81 ± 0.02	10588.75 ± 655.21
4	7/30/2023	21.76 ± 0.03	7.28 ± 0.02	10411.38 ± 532.86
4	7/31/2023	21.73 ± 0.02	7.64 ± 0.01	5488.66 ± 255.59
5	7/14/2023	21.21 ± 0.11	8.66 ± 0.05	10772 ± 710.78
5	7/15/2023	20.52 ± 0.01	8.68 ± 0.01	8629.34 ± 477.51
5	7/16/2023	20.01 ± 0.02	8.65 ± 0.01	8631.15 ± 578.47
5	7/17/2023	19.82 ± 0.01	8.68 ± 0.01	6712.38 ± 437.32
5	7/18/2023	20 ± 0.03	8.71 ± 0.01	11190.04 ± 602.21
5	7/19/2023	20.13 ± 0.01	8.78 ± 0.01	7587.34 ± 521.39
5	7/20/2023	20.79 ± 0.05	8.76 ± 0.01	15706.13 ± 935.73
5	7/21/2023	21.27 ± 0.02	8.84 ± 0	12693.22 ± 763.52
5	7/22/2023	21.8 ± 0.05	8.81 ± 0.01	14631.49 ± 815.26
5	7/23/2023	21.67 ± 0.02	8.87 ± 0.01	8838.13 ± 434.66

5	7/24/2023	21.8 ± 0.04	8.79 ± 0	18131.28 ± 956.21
5	7/25/2023	21.62 ± 0.01	8.79 ± 0	5585.75 ± 314.13
5	7/26/2023	22.38 ± 0.05	8.69 ± 0.01	16616.7 ± 934.93
5	7/27/2023	22.93 ± 0.03	8.58 ± 0	15022.32 ± 837.92
5	7/28/2023	22.26 ± 0.02	8.46 ± 0.01	6821.37 ± 347.49
5	7/29/2023	21.72 ± 0.02	8.5 ± 0.01	12630.88 ± 738.48
5	7/30/2023	21.77 ± 0.03	8.58 ± 0.01	12894.42 ± 628.88
5	7/31/2023	21.73 ± 0.02	8.62 ± 0.01	7217.95 ± 337.46
6	7/14/2023	21.23 ± 0.11	8.83 ± 0.05	6925.28 ± 380.48
6	7/15/2023	20.59 ± 0.01	8.77 ± 0.01	6929.56 ± 351.97
6	7/16/2023	20.06 ± 0.01	8.71 ± 0.01	7258.43 ± 462.24
6	7/17/2023	19.88 ± 0.01	8.71 ± 0.01	5659.12 ± 322.27
6	7/18/2023	20.03 ± 0.03	8.68 ± 0.01	9415.71 ± 493.08
6	7/19/2023	20.18 ± 0.01	8.74 ± 0.01	6361.32 ± 427.5
6	7/20/2023	20.83 ± 0.05	8.69 ± 0.01	11860.95 ± 693.1
6	7/21/2023	21.29 ± 0.02	8.77 ± 0.01	7564.47 ± 441.34
6	7/22/2023	21.8 ± 0.04	8.7 ± 0.01	9905.02 ± 512.99
6	7/23/2023	21.72 ± 0.02	8.77 ± 0.01	7473.39 ± 339.19
6	7/24/2023	21.79 ± 0.03	8.66 ± 0.01	10863.07 ± 599.49
6	7/25/2023	21.66 ± 0.01	8.64 ± 0	3928.42 ± 202.7
6	7/26/2023	22.38 ± 0.05	8.46 ± 0.01	10939.62 ± 618.29
6	7/27/2023	22.95 ± 0.03	8.45 ± 0	7917.44 ± 389.3
6	7/28/2023	22.31 ± 0.01	8.37 ± 0.01	5142.48 ± 254.01
6	7/29/2023	21.77 ± 0.02	8.39 ± 0.01	8670.41 ± 534.96
6	7/30/2023	21.81 ± 0.03	8.39 ± 0.01	7876.48 ± 399.82
6	7/31/2023	21.74 ± 0.02	8.37 ± 0.01	4834.66 ± 223.05
7	7/14/2023	21.33 ± 0.11	8.42 ± 0.05	7207.81 ± 438.51
7	7/15/2023	20.6 ± 0.01	8.41 ± 0.01	6930.64 ± 383.17
7	7/16/2023	20.09 ± 0.02	8.39 ± 0.01	6745.74 ± 459.03
7	7/17/2023	19.92 ± 0.01	8.44 ± 0.01	5575.5 ± 353.39
7	7/18/2023	20.08 ± 0.03	8.45 ± 0	8605.81 ± 421.51
7	7/19/2023	20.21 ± 0.02	8.5 ± 0	5469.91 ± 338.21
7	7/20/2023	20.87 ± 0.05	8.51 ± 0.01	10301.52 ± 535.37
7	7/21/2023	21.3 ± 0.02	8.59 ± 0.01	6847.91 ± 378.52
7	7/22/2023	21.82 ± 0.04	8.54 ± 0.01	8748.08 ± 411.33
7	7/23/2023	21.73 ± 0.02	8.62 ± 0.01	7582.07 ± 341.06
7	7/24/2023	21.79 ± 0.04	8.23 ± 0.02	11883.36 ± 666.9
7	7/25/2023	21.66 ± 0.01	8.57 ± 0	5144.14 ± 298.21
7	7/26/2023	22.4 ± 0.05	8.43 ± 0.01	14665.41 ± 794.11
7	7/27/2023	22.97 ± 0.03	8.39 ± 0	13016.68 ± 746.74
7	7/28/2023	22.33 ± 0.02	8.26 ± 0.01	5197.53 ± 252.93
7	7/29/2023	21.74 ± 0.02	8.31 ± 0.01	10844.37 ± 677.8

7	7/30/2023	21.79 ± 0.03	8.37 ± 0.01	11469.34 ± 570.54
7	7/31/2023	21.77 ± 0.02	8.43 ± 0.01	5859.07 ± 276.1
8	7/14/2023	21.27 ± 0.03	8.34 ± 0.02	8256.73 ± 486.22
8	7/15/2023	20.53 ± 0.01	8.29 ± 0.01	7246.2 ± 412.82
8	7/16/2023	20.05 ± 0.02	8.27 ± 0.01	7508.13 ± 524.3
8	7/17/2023	19.87 ± 0.01	8.24 ± 0.01	5497.17 ± 339.5
8	7/18/2023	20.05 ± 0.03	8.06 ± 0.01	9215.89 ± 475.55
8	7/19/2023	20.15 ± 0.02	8.23 ± 0.01	5968.58 ± 393.12
8	7/20/2023	20.85 ± 0.05	8.28 ± 0.01	12639.99 ± 709.31
8	7/21/2023	21.25 ± 0.02	8.26 ± 0.01	9162.86 ± 533.58
8	7/22/2023	21.82 ± 0.05	8.11 ± 0.01	11513.95 ± 606.67
8	7/23/2023	21.67 ± 0.02	8.31 ± 0.01	8294.9 ± 383.63
8	7/24/2023	21.76 ± 0.04	8.21 ± 0.01	13287.43 ± 749.38
8	7/25/2023	21.59 ± 0.01	8.12 ± 0.01	4951.02 ± 251.82
8	7/26/2023	22.36 ± 0.05	8.08 ± 0.01	15257.3 ± 815.72
8	7/27/2023	22.88 ± 0.03	7.92 ± 0.01	12294.97 ± 679.16
8	7/28/2023	22.23 ± 0.02	7.92 ± 0.02	6110.59 ± 300.59
8	7/29/2023	21.68 ± 0.02	8.02 ± 0.01	9129.77 ± 547.05
8	7/30/2023	21.74 ± 0.03	7.99 ± 0.01	10202.85 ± 521.78
8	7/31/2023	21.71 ± 0.02	7.95 ± 0.01	5748.26 ± 264.09
